# A cluster of noncoding RNAs activates the *ESR1* locus during breast cancer adaptation

**DOI:** 10.1038/ncomms7966

**Published:** 2015-04-29

**Authors:** Saori Tomita, Mohamed Osama Ali Abdalla, Saori Fujiwara, Haruka Matsumori, Kazumitsu Maehara, Yasuyuki Ohkawa, Hirotaka Iwase, Noriko Saitoh, Mitsuyoshi Nakao

**Affiliations:** 1 Department of Medical Cell Biology, Institute of Molecular Embryology and Genetics, Kumamoto University, Kumamoto 860-0811, Japan; 2 Department of Breast and Endocrine Surgery, Graduate School of Medical Sciences, Kumamoto University, Kumamoto 860-8556, Japan; 3 Department of Advanced Initiative Medicine, Faculty of Medicine, Kyushu University, Fukuoka 812-8582, Japan; 4 Program for Leading Graduate Schools ‘HIGO (Health life science: Interdisciplinary and Glocal Oriented) Program', Kumamoto University, Kumamoto 860-8556, Japan; 5 Core Research for Evolutional Science and Technology (CREST), Japan Science and Technology Agency, Tokyo 102-0076, Japan

## Abstract

Estrogen receptor-α (ER)-positive breast cancer cells undergo hormone-independent proliferation after deprivation of oestrogen, leading to endocrine therapy resistance. Up-regulation of the ER gene (*ESR1*) is critical for this process, but the underlying mechanisms remain unclear. Here we show that the combination of transcriptome and fluorescence *in situ* hybridization analyses revealed that oestrogen deprivation induced a cluster of noncoding RNAs that defined a large chromatin domain containing the *ESR1* locus. We termed these RNAs as *Eleanors* (ESR1 locus enhancing and activating noncoding RNAs). *Eleanors* were present in ER-positive breast cancer tissues and localized at the transcriptionally active *ESR1* locus to form RNA foci. Depletion of one *Eleanor*, *upstream* (*u)-Eleanor*, impaired cell growth and transcription of intragenic *Eleanors* and *ESR1* mRNA, indicating that *Eleanors cis-*activate the *ESR1* gene. *Eleanor*-mediated gene activation represents a new type of locus control mechanism and plays an essential role in the adaptation of breast cancer cells.

## Abstract (web summary)

Estrogen-receptor-positive breast cancer cells undergo hormone-independent proliferation after long-term oestrogen deprivation and become resistant to endocrine therapies. Here, the authors report a cluster of noncoding RNAs important for this adaptation process.

## Graphical abstract

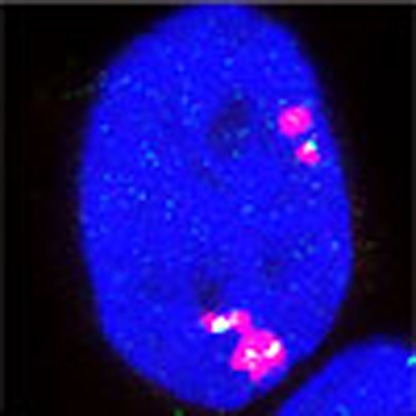

Cancer cells adapt to the surrounding environment and maintain their proliferation, resulting in malignant transformation and resistance to anticancer treatments^1^. Breast cancers expressing estrogen receptor-α (ER) depend on oestrogen for cellular growth and survival. ER functions as a nuclear receptor-type transcription factor upon binding to oestrogen and regulates the expression of various target genes. Endocrine therapies, such as the use of an aromatase inhibitor (AI) that blocks oestrogen production, are the most effective for ER-positive breast cancers^2^. However, these treatments are frequently followed by disease recurrence because most breast tumours, which are initially responsive to these therapies, develop resistances through unknown mechanisms^2^,^3^,^4^.

MCF7 human breast cancer cells are ER-positive and acquire oestrogen-independent proliferation when they are cultured under an oestrogen-depleted condition for a prolonged period of time (long-term oestrogen deprivation; LTED)^5^,^6^. LTED adaptation is a well-established cellular model that recapitulates acquisition of AI resistance or postmenopausal tumorigenesis^5^,^6^,^7^,^8^,^9^,^10^. Previous studies have reported that the gene-encoding ER (*ESR1*) is up-regulated during LTED adaptation^8^, which is found in ER-positive human breast cancers. Understanding the molecular mechanism of this gene activation is critical because overproduction of ER may lead to an enhanced response to low concentrations of oestrogen, which is responsible for the LTED-adapted phenotype^7^,^9^,^11^. Paradoxically, administration of oestrogen is an effective treatment for AI-resistant breast cancers^12^,^13^,^14^, and the LTED cell model may be used to gain the mechanistic evidence for such therapeutic efficacy.

Gene expression patterns are reprogrammed in response to environmental changes or during development and linked to the conversion of cellular phenotypes. Several events that occur in chromatin include recruitment of transcriptional activators/repressors, changes in histone/DNA modifications, RNA polymerase II (RNA Pol II) binding, long-range chromosomal interactions and chromatin domain formation^15^,^16^,^17^,^18^. It was classically shown that the *β-globin* locus forms a distinct open chromatin domain during erythropoiesis^16^,^17^,^18^. To date, various types of chromatin domains have been characterized as ∼10 kb to a few Mb in length by genome-wide chromosome conformation, histone modification patterns, association with specific nuclear architectures and nuclease sensitivities^19^,^20^,^21^,^22^. These data suggest that interphase chromosomes are organized by hierarchical folding through which transcription can be regulated through chromatin domain formation.

Recent studies have revealed that noncoding RNAs (ncRNAs) are also involved in transcriptional regulation through diverse functions^23^. The mammalian transcriptome includes thousands of long noncoding RNAs (lncRNAs) that are longer than 200 nucleotides and devoid of protein-coding potential^24^. Some lncRNAs show unique expression under specific conditions such as X chromosome inactivation, genomic imprinting and maintenance or differentiation of stem cells^25^,^26^,^27^. LncRNAs are encoded at virtually any site of the genome, including enhancer, promoter, intron and intergenic regions, which regulate genes both in *cis* and *trans*. Currently, the potential roles of lncRNAs in cancer cell adaptation are unknown.

In the present study, we found that up-regulation of *ESR1* was important for LTED cell adaptation, which was maintained by novel ncRNAs produced from a large chromatin domain of the *ESR1* gene. Fluorescence *in situ* hybridization (FISH) analyses showed that these ncRNAs, termed *Eleanors* (ESR1 locus enhancing and activating noncoding RNAs), were localized at the site of active transcription, resulting in the formation of distinct RNA foci in the nucleus. One of the *Eleanors*, *upstream-Eleanor* (*u-Eleanor*), originated from an enhancer-like sequence upstream of the *ESR1* gene, which was necessary for enhanced expression of both *ESR1* mRNA and intragenic *Eleanors* in LTED cells. Our genome-wide transcriptome analyses revealed that coordinated expression of ncRNA and mRNA, exemplified by the *ESR1* gene, was conserved in a set of long genes. These findings uncover the molecular basis for endocrine therapy-resistant breast cancer, which involves a new type of ncRNA-mediated regulation of a chromatin domain and protein-coding genes.

## Results

### *ESR1* up-regulation is accompanied by *Eleanor* expression

**Figure 1:**
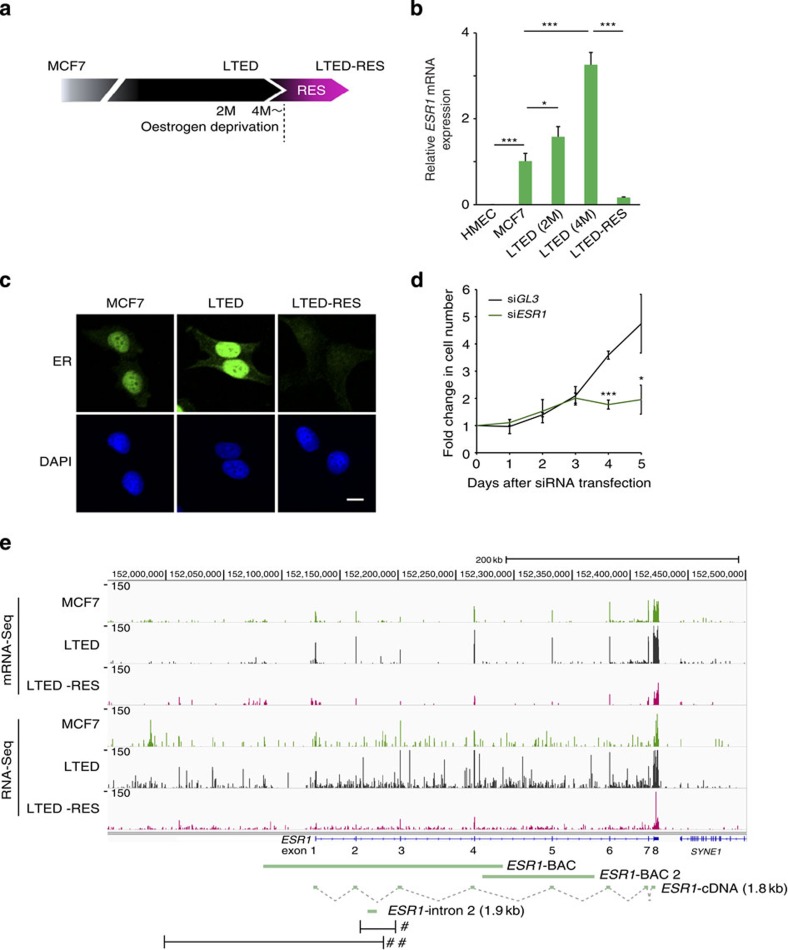
*Eleanors* and *ESR1* mRNA are coordinately expressed in LTED and LTED-RES cells. (**a**) Schematic representation of the cell models used in this study. ER-positive MCF7 breast cancer cells were cultured under three conditions: MCF7, LTED and LTED-RES. (**b**) Expression levels of *ESR1* mRNA. qRT–PCR results under the MCF7 condition were set to 1. Primers were designed to cover the exon–exon junction. Values are the means±s.d.; *n*=3. *P*-values were calculated using Student's *t*-test (*P<0.05, ***P<0.001). (**c**) Immunofluorescence of ER showing enhanced expression in LTED cells and its suppression by resveratrol treatment (LTED-RES). Scale bar, 10 μm. (**d**) *ESR1* knockdown inhibits LTED cell proliferation. LTED cells were treated with siRNA targeting *ESR1* for the indicated periods. Cell growth is shown as fold changes. Values are the means±s.d.; *n*=3. *P*-values were calculated using Student's *t*-test (*P<0.05, ***P<0.001). (**e**) Gene tracks representing mRNA-Seq and RNA-Seq data of the human *ESR1* locus. Novel ncRNAs, termed *Eleanors*, were abundantly expressed in LTED cells from the entire *ESR1* locus, which were detected as read signals in non-exonic regions. *Eleanors* were suppressed in LTED-RES cells. The structures of *ESR1* and downstream *SYNE1* genes are shown below. Green bars indicate the FISH probes used in this study. Regions highlighted in Figs 2a and 4a are denoted by # and ##, respectively. 2M, two months; 4M, four months.

To understand the mechanism of hormonal adaptation and the action of resveratrol in ER-positive breast cancers, we used a cell model system in which MCF7 cells were cultured under three different conditions: normal (MCF7), oestrogen deprivation for 2–4 months (LTED) and further treatment with 100 μM resveratrol for 24 h (LTED-RES, Fig. 1a). Resveratrol is structurally similar to oestrogen, binds to ER *in vitro* and exerts oestrogenic effects on breast cancer cells^28^,^29^. Quantitative PCR with reverse transcription (qRT–PCR) and immunofluorescence analyses showed that *ESR1* expression was significantly increased in LTED cells and dramatically suppressed by resveratrol (Fig. 1b,c). Notably, knockdown of ER significantly reduced LTED cell proliferation at 96 h after transfection of the small interfering RNA (siRNA) (Fig. 1d). This result suggests that the up-regulation of ER plays a role in acquisition of oestrogen-independent cancer cell growth.

To further investigate activation of the *ESR1* gene, we performed mRNA-Seq and RNA-Seq analyses of cells under the three conditions. We prepared poly (A)^+^ RNA for mRNA-Seq, and total RNA that was devoid of ribosomal RNA for RNA-Seq, respectively (see Methods for details). Gene tracks representing mRNA-Seq and RNA-Seq data are shown in Fig. 1e. The human *ESR1* locus resides on chromosome 6, consists of eight exons and is ∼300 kb in length. As expected, mRNA-Seq data showed up-regulation of *ESR1* exons in LTED cells and repression in LTED-RES cells (Fig. 1e, top three tracks). Interestingly, RNA-Seq analyses detected a significant amount of intragenic transcripts in LTED cells, which extended along the entire *ESR1* locus including introns and upstream noncoding regions, but not to the neighbouring silent gene, *SYNE1* (Fig. 1e, fifth track). We named the noncoding RNAs produced from inside and around the *ESR1* locus (6q25.1; 152083078–152424447) as ‘*Eleanors*'. Production of *Eleanors* was well synchronized with production of *ESR1* mRNA, suggesting that *Eleanors* participate in regulation of the *ESR1* gene. *Eleanors* were distinct from previously reported types of ncRNAs, such as enhancer/promoter-RNAs and miRNAs, because *Eleanors* were expressed from a much larger chromatin region.

### *Eleanors* are localized at the site of *ESR1* transcription

**Figure 2:**
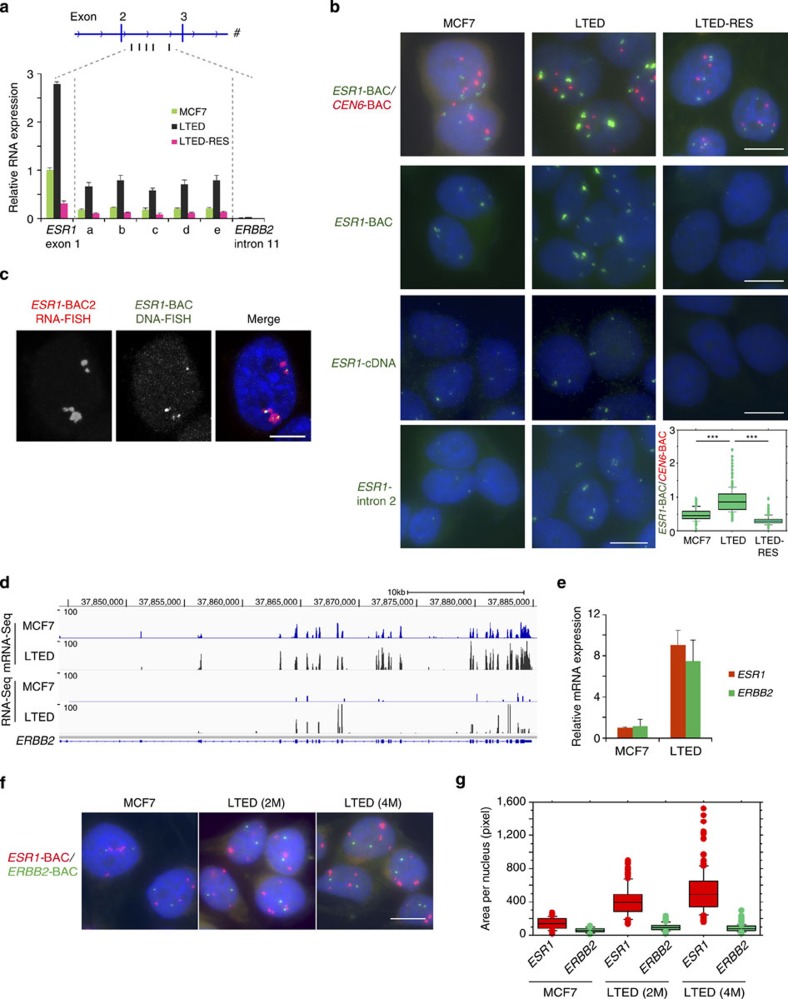
*Eleanors* are associated with the site of *ESR1* transcription in the nucleus. (**a**) Expression of *Eleanor* transcripts from various sites (**a**–**e**) within intron 2 of the *ESR1* gene. *ESR1* exon 1 and *ERBB2* intron 11 were used as controls. For qRT–PCR, total RNA was pre-treated with DNase I. The value for *ESR1* exon 1 in MCF7 cells was set to 1. Values are the means±s.d.; *n*=3. Corresponding ΔC_t_ values are listed in Supplementary Table 6. (**b**) *Eleanor* FISH signals were enlarged in LTED cells and diminished by resveratrol treatment (LTED-RES). The BAC probes covered the *ESR1* locus (*ESR1*-BAC), a centromeric region of human chromosome 6 (*CEN6*-BAC), exons only (*ESR1*-cDNA) and the intron only (*ESR1-*intron 2, see Fig. 1e, bottom). Cellular DNA was heat-denatured in the top panels, but not in others. *Eleanor* foci were enlarged in LTED cells, which were detected with *ESR1*-BAC and intron probes, but not the *ESR1*-cDNA probe. Box plots on the bottom right show quantification of FISH signals in the top panels (*n*>170 nuclei for each sample). *P*-values were calculated using Student's *t*-test (***P<0.001). Scale bar, 10 μm. (**c**) Sequential hybridization to *Eleanor* ncRNAs (RNA FISH, left) and the *ESR1* gene (DNA FISH, middle) showing co-localization (right). Cellular RNAs were digested with RNase before DNA hybridization. Scale bar, 5 μm. (**d**) Gene tracks representing mRNA-Seq and RNA-Seq data of the *ERBB2* locus in MCF7 and LTED cells. Compared with the *ESR1* locus (Fig. 1e), there was no induction of ncRNAs in the *ERBB2* locus of LTED cells. (**e**) Comparable induction levels of *ESR1* and *ERBB2* mRNAs in LTED cells. qRT–PCR results under the MCF7 condition were set to 1. Values are the means±s.d.; *n*=3. Corresponding ΔC_t_ values are listed in Supplementary Table 6. (**f**) Representative FISH images using BAC probes for the *ESR1* locus (red) and *ERBB2* locus (green). *ERBB2* signals were not enlarged in LTED cells. Scale bar, 10 μm. (**g**) Box plot showing quantification of FISH data in **f** (*n*>100 nuclei for each). 2M, two months; 4M, four months.

To confirm the presence of *Eleanors*, we assessed transcripts from *ESR1-*intron 2 by qRT–PCR (Fig. 2a). Generally, introns are rapidly processed to undetectable levels after transcription as shown for intron 11 of *ERBB2*. However, transcripts from intron 2 of *ESR1* were unusually stable in LTED cells. To further examine the presence of ncRNAs derived from the broad region of the *ESR1* locus, we performed FISH analyses using bacterial artificial chromosome (BAC) probes that covered most of the *ESR1* locus (*ESR1*-BAC) (Figs 1e and 2b). First, cells were processed for DNA FISH using the BAC clone for the centromeric region of chromosome 6 (*CEN6-*BAC) as a control (Fig. 2b, top panels). We detected three or four *CEN6* signals (red) in the nucleus because of the standard karyotypes of MCF7 cells^30^. As expected, *ESR1* signals (green) were detected close to each *CEN6* signal. However, it was surprising that the *ESR1* signals were significantly larger in LTED cells (∼2-fold higher *ESR1/CEN6* area ratio compared with that in MCF7 cells) and obviously smaller in LTED-RES cells (Fig. 2b, bottom right panel).

The enlarged FISH signals may suggest homogeneously staining regions that are cytogenetic hallmarks of genomic amplification in cancer^31^. However, such a notion was not the case for the *ESR1* locus in LTED cells, because the enlarged foci were promptly reduced to small dots by resveratrol treatment. Indeed, we found no *ESR1* gene amplification in copy number variation analysis (Supplementary Fig. 1a,b). Further analyses showed that most of the enlarged FISH signals consisted of RNA molecules, because they were significantly diminished by treatment with RNase, but not DNase (Supplementary Fig. 1c). To clarify the origin of the RNAs accumulating in the enlarged foci, we performed FISH under non-denaturing conditions (RNA FISH) using the *ESR1-*BAC probe (covering noncoding regions as well as exons), *ESR1*-cDNA probe (covering exons exclusively) and *ESR1*-intron 2 probe (Fig. 2b lower three panels). The enlarged signals in LTED cells were detected with the BAC and intron 2 probes, but not the *ESR1-*cDNA probe, indicating that ncRNAs derived from the intragenic region (*Eleanors*) were the major components of the enlarged FISH signals.

We investigated the spatial positioning of *Eleanors* relative to the *ESR1* locus in the LTED nucleus by sequential hybridizations. First, *Eleanors* were hybridized with the *ESR1*-BAC2 probe (shown in Fig. 1e), followed by fixation of the signals and subsequent RNase treatment, and then the *ESR1* gene was hybridized with the *ESR1*-BAC probe. The results showed that *Eleanors* and the *ESR1* locus were co-localized in the nucleus (Fig. 2c). Collectively, these data demonstrate that *Eleanors* are produced from the intragenic region of the transcriptionally active *ESR1* locus in LTED cells and remain associated with the site of transcription, resulting in the formation of distinct RNA foci. Our stranded RNA-Seq results indicated that *Eleanors* were transcribed in the same direction as that of *ESR1* mRNA.

To investigate whether ncRNA production in LTED cells is specific to the *ESR1* locus, we examined the *ERBB2* gene that plays a role in a subset of breast cancers. Gene tracks representing mRNA-Seq and RNA-Seq data of the *ERBB2* locus are shown in Fig. 2d. *ERBB2* gene expression was not accompanied by ncRNA production in LTED cells. However, similar to *ESR1* expression, *ERBB2* expression was activated by more than 3-fold (Fig. 2e). Consistently, FISH signals for the *ERBB2* locus (green) showed no changes, while signals for the *ESR1* locus (red) were large in the same nucleus (Fig. 2f,g). Two other genes, *APP* and *ERGIC*, also showed similar levels of up-regulation in LTED cells without significant RNA expression from noncoding regions (Supplementary Fig. 2).

Adaptation to hormone deprivation is conserved among several breast cancer cells. Another ER-positive breast cancer cell line, HCC1428 acquires oestrogen-independent proliferation with up-regulation of ER after a long period of oestrogen deprivation (HCC1428 LTED cells)^32^. Using an *ESR1*-BAC probe, we detected the enlarged FISH signals in HCC1428 LTED cells, which were suppressed by resveratrol treatment (HCC1428 LTED-RES cells), similar to LTED and LTED-RES cells (Supplementary Fig. 1d).

Together, the *Eleanor*-containing foci were characteristic of the *ESR1* locus in LTED cells, suggesting a new type of gene activation mechanism for the specific gene locus.

### *Eleanors* are present in ER-positive breast cancer tissues

**Figure 3:**
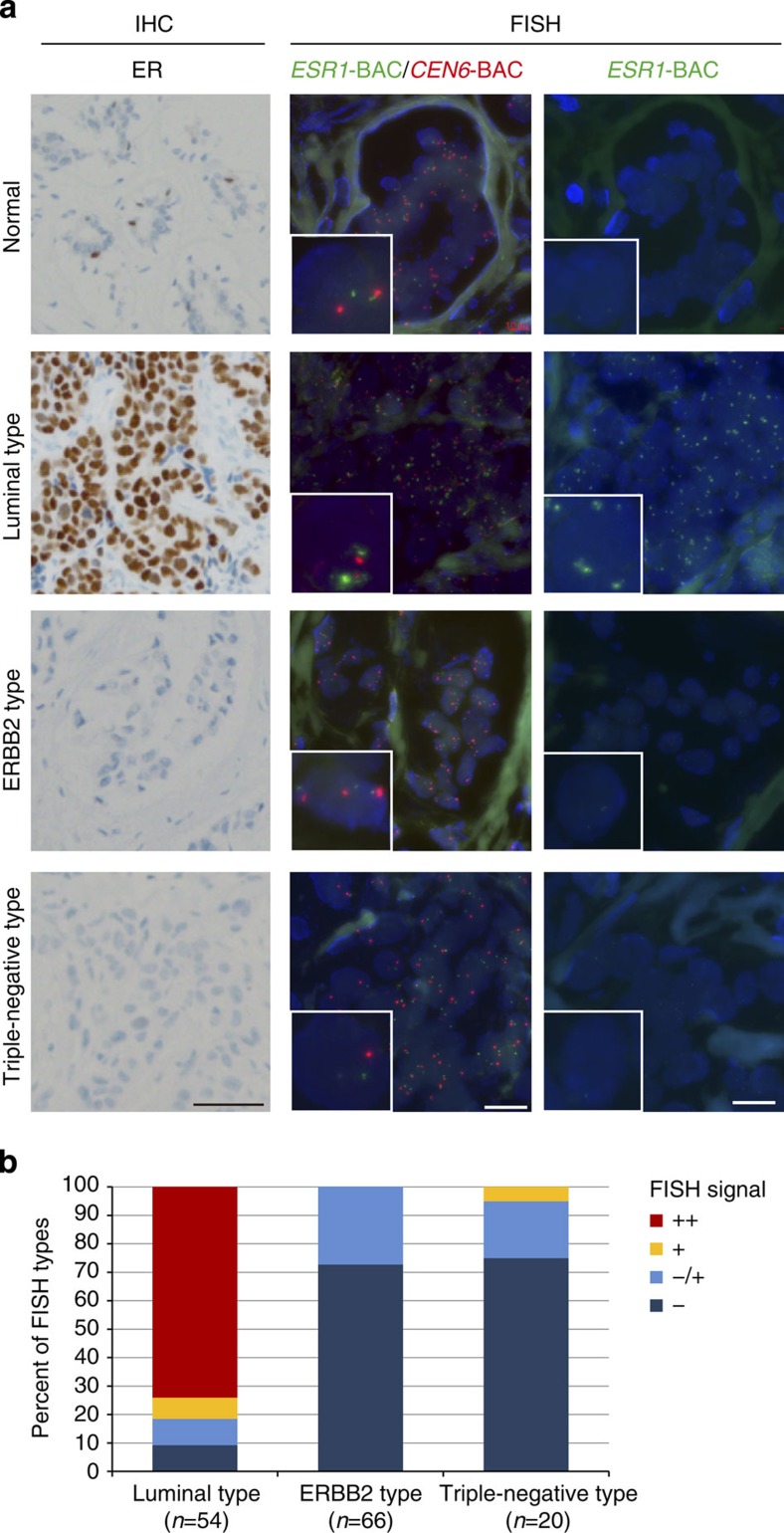
*Eleanor* expression in ER-positive breast cancer tissues. (**a**) IHC and FISH analyses of breast cancer tissues. Serial sections of breast cancer tissues with the indicated types were subjected to IHC using an anti-ER antibody (IHC, left) and FISH using BAC probes for *ESR1* and *CEN6* (middle and right, respectively). Large *Eleanor*-containing foci were detected in the luminal type (ER positive). The DNA was processed with or without heat denaturation (middle and right, respectively). Scale bars, 50 μm (left) and 20 μm (middle and right). (**b**) Summary of FISH analyses of breast cancer patients. Strong FISH signals (++) were exclusively present in a subset of ER-expressing breast cancers (luminal type). Detailed data are provided in Supplementary Table 4.

Naturally occurring human breast cancers are grouped into at least three subtypes: a luminal type that is ER-positive, an ERBB2 type that highly expresses ERBB2 with genomic amplifications, and a triple-negative type that is negative for ER, ERBB2 and the progesterone receptor^33^,^34^. To confirm the appearance of *Eleanor*-containing foci *in vivo*, we performed a combination of immunohistochemistry (IHC) and FISH analyses using serial sections of various breast cancer tissues (Fig. 3). We detected the enlarged FISH signals for *Eleanors* (green) in some luminal-type breast cancers, which were well correlated to ER overexpression found in IHC. On the other hand, these FISH signals were absent from normal breast tissue as well as ERBB2-type and triple-negative-type cancer tissues, all of which were ER negative. These results suggest significant implications of *Eleanors* in ER-positive breast cancer cells.

### *u-Eleanor* enhances intragenic *Eleanor* and *ESR1* mRNA

**Figure 4:**
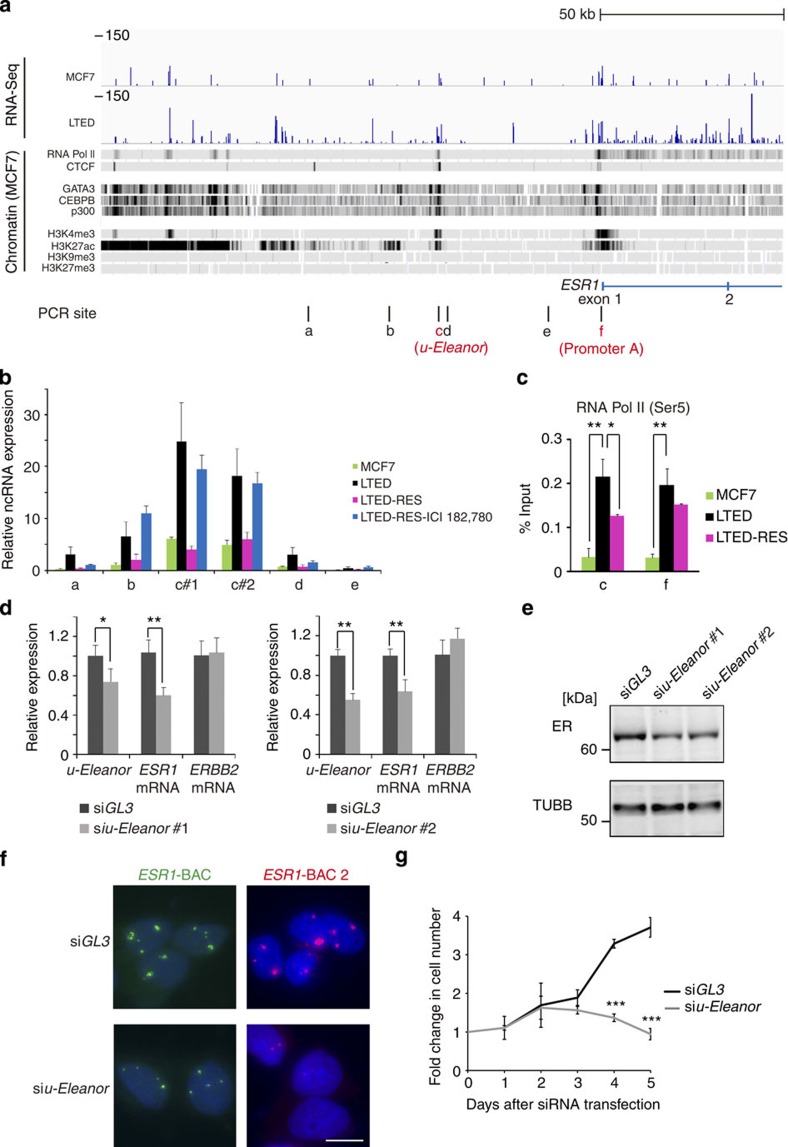
*u-Eleanor* plays a role in coordinated up-regulation of intragenic *Eleanor* and *ESR1* mRNA to promote the proliferative activity of LTED cells. (**a**) Overview of a region upstream of the *ESR1* locus. The RNA-Seq tracks were aligned with the ChIP-Seq data available in the UCSC genome browser^39^ (University of California, Santa Cruz, CA, and Supplementary Table 1). Sites amplified by qPCR are shown (**a**–**f**). (**b**) Local expression of *u-Eleanor*. *u-Eleanor* was induced at site c in LTED cells, repressed in LTED-RES cells, and de-repressed by ICI 182,780 treatment (ER antagonist, related to Fig. 5e–g). For qRT–PCR, total RNA was pre-treated with DNase I, and the amplification efficiency for each primer set was normalized. The value for site b in MCF7 was set to 1. Values are the means±s.d.; *n*=3. Corresponding ΔC_t_ values are listed in Supplementary Table 6. (**c**) RNA Pol II binding to the *u-Eleanor* region (**c**) and *ESR1* promoter A (f). For ChIP-qPCR, values are the means±s.d.; *n*=3. *P*-values were calculated using Student's *t*-test (**P<0.01). (**d**) Decreased expression of *ESR1* mRNA upon *u-Eleanor* knockdown by specific siRNAs. Expression levels of *ESR1* and *ERBB2* mRNAs were measured by qRT–PCR. Values are the means±s.d.; *n*=3. *P*-values were calculated using Student's *t*-test (*P<0.05, **P<0.01). (**e**) Reduced protein level of ER upon *u-Eleanor* knockdown. An immunoblot of ER is shown. TUBB served as a loading control. (**f**) Reduction of *Eleanor*-containing foci by *u-Eleanor* knockdown in LTED cells. Independent BAC probes shown in Fig. 1e were used for the FISH analyses. Scale bar, 10 μm. (**g**) Inhibition of LTED cell proliferation by *u-Eleanor* knockdown. LTED cells were treated with siRNA targeting *u-Eleanor* for the indicated periods. Cell numbers are shown as fold changes. Values are the means±s.d.; *n*=3. *P*-values were calculated using Student's *t*-test (*P<0.05, ***P<0.001).

Our RNA-Seq data showed that the region producing ncRNAs extended further upstream of the *ESR1* gene (Fig. 4a). One of the peaks on the gene track was positioned at site c that was ∼40 kb upstream of the canonical promoter A (site f) in MCF7 cells^35^,^36^. qRT–PCR analyses showed local transcription from site c (Fig. 4b). Stranded RNA-Seq data indicated that *u-Eleanor* was transcribed in the same direction as that of intragenic *Eleanors* and *ESR1* mRNA. In agarose gel electrophoresis of RT–PCR products, we detected a transcript of at least 1,200 nucleotides in length (Supplementary Fig. 3a,b). We termed this lncRNA as *u-Eleanor*.

Alignment with the chromatin immunoprecipitation-sequencing (ChIP-Seq) data revealed that the *u-Eleanor* gene region (site c) was bound by RNA Pol II, CTCF and transactivators (GATA3, CEBPB and p300) in MCF7 cells (Supplementary Table 1). Consistently, this site was enriched with active histone marks (trimethyl-H3K4 and acetyl-H3K27) and devoid of repressive marks (trimethyl-H3K9 and trimethyl-H3K27). These active chromatin features at the *u-Eleanor* gene region were characteristic of cell types of breast origin. Similar to enhancer RNAs (eRNAs), these data suggest that site c functions as an upstream regulatory element that is actively transcribed into ncRNAs^37^.

Alternatively, *u-Eleanor* could be an unannotated upstream promoter of *ESR1*. Although promoter A is most frequently used in MCF7 cells^36^, transcription of *ESR1* can be complex under certain circumstances because of the nature of differential promoter usage^35^,^36^. Gene annotations in UCSC and GENCODE genome browsers^38^,^39^ revealed a variety of cDNAs and ESTs, including independent transcripts, which terminate before the *ESR1* gene, as well as a transcript that spans from the upstream region to the complete body of *ESR1* (Supplementary Fig. 3a). However, *u-Eleanor* did not correspond to any of the previously described alternative promoters^35^,^36^. *u-Eleanor* was unlikely to be contiguous with the downstream *Eleanors* because we did not detect any transcripts from sites d or e (Fig. 4b and Supplementary Fig. 3a,b). In addition, RT–PCR using several primer sets starting in *u-Eleanor* and ending in *ESR1* exon 1 failed to detect any transcripts (cg in Supplementary Fig. 3b). Furthermore, no protein-coding possibility was predicted in the *u-Eleanor* gene region (Supplementary Table 5). Taken together, we conclude that *u-Eleanor* is a transcript independent of intragenic *Eleanors* and *ESR1* mRNA.

Transcription of *u-Eleanor* may enhance *ESR1* mRNA expression, because both RNAs were up-regulated in LTED cells and repressed in LTED-RES cells (Figs 1b and 4b). We suspected that *u-Eleanor* might function as an eRNA, which was in agreement with our ChIP-qPCR data showing that the *u-Eleanor* region and promoter A of *ESR1* were bound by the active form of RNA Pol II (phosphorylated at serine 5) in LTED cells (Fig. 4c). In addition, *u-Eleanor* chromatin was enriched with mono-methylation of H3K4 (H3K4me1) rather than tri-methylation (H3K4me3) (Supplementary Fig. 3c,d), suggesting that this region functions as an enhancer^40^,^41^. On the other hand, promoter A was high in H3K4me3 relative to H3K4me1. The aligned ChIP-Seq data showed that the *u-Eleanor* region was also bound by CTCF, an organizer for the three-dimensional structure of the genome. Chromosome conformation capture experiments suggested that the *u-Eleanor* region as well as exon 1 and intron 1 of the *ESR1* gene were close together in LTED cells compared with that in MCF7 and LTED-RES cells (Supplementary Fig. 4). These results suggest that the upstream chromatin structure of this locus is significantly altered during LTED adaptation.

We tested whether *u-Eleanor* is involved in transcriptional activation of the *ESR1* locus in LTED cells. Upon reduction of *u-Eleanor* to 50–70% by two independent siRNAs, *ESR1* mRNA expression was decreased to ∼60% (*P*<0.01, *P*-values; Student's *t*-test ) without affecting *ERBB2* mRNA (Fig. 4d). Consistently, the ER protein level was decreased with the reduction of *u-Eleanor* (Fig. 4e). Furthermore, FISH analyses of LTED cells were performed using *ESR1*-BAC and *ESR1*-BAC2 (shown in Fig. 1e) as independent probes (Fig. 4f). *u-Eleanor* knockdown efficiently diminished the enlarged FISH signals, indicating that *u-Eleanor* maintains downstream intragenic *Eleanor* in LTED cells.

Next, we examined the effect of *Eleanors* on the proliferative activity of LTED cells. As a result, the cell number was significantly reduced at 96 h after knockdown of *u-Eleanor* by siRNA (Fig. 4g). Thus, *u-Eleanor* has an essential role in the enhanced transcription of *Eleanors* and mRNA from the *ESR1* locus, as well as the cell proliferation and viability during LTED adaptation.

### Resveratrol exerts a repressive effect on *Eleanor* via ER

**Figure 5:**
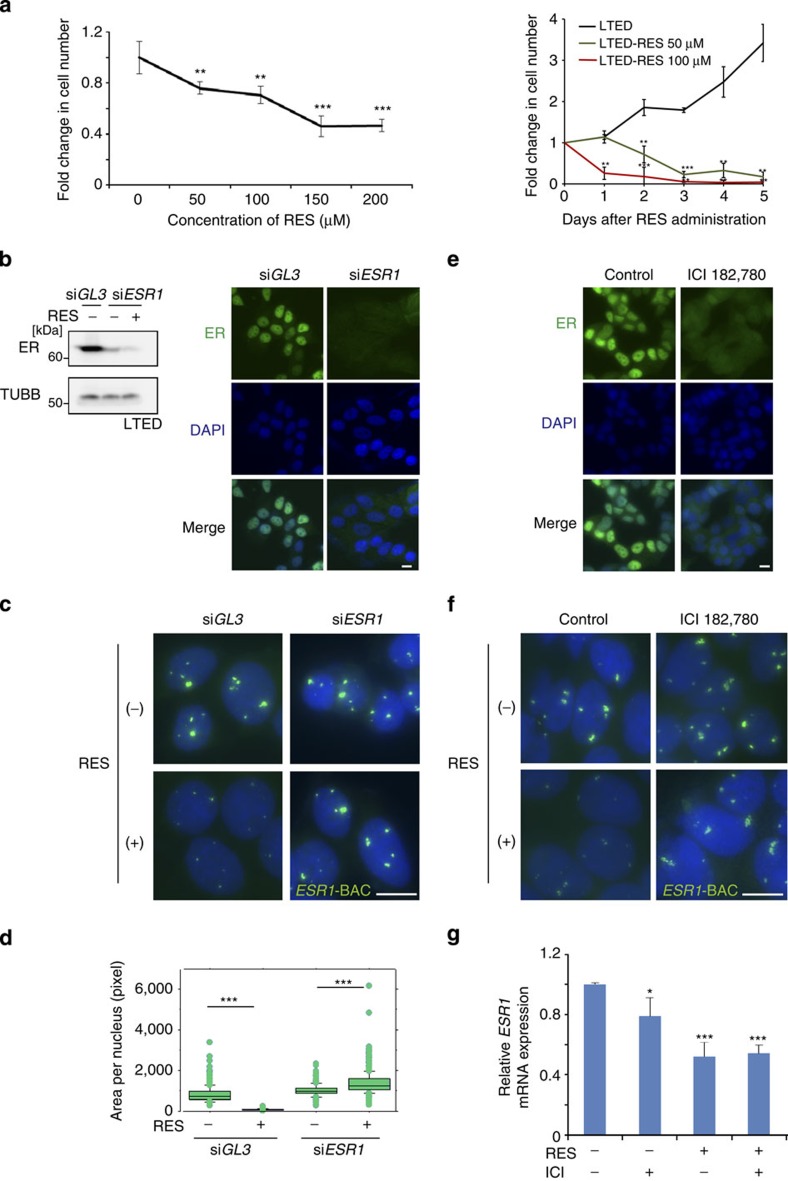
Resveratrol exerts a repressive effect on *Eleanors* via ER. (**a**) Inhibition of LTED cell proliferation by resveratrol treatment. Cell proliferation was inhibited in a dose-dependent manner (left). The number of control LTED cells was set to 1. A time course analysis was performed using 50 and 100 μM resveratrol (right). The number of LTED cells at day 0 was set to 1. Values are the means±s.d.; *n*=3. *P*-values were calculated using Student's *t*-test (**P<0.01, ***P<0.001). (**b**) Immunoblot and immunofluorescence analyses of siRNA-mediated ER knockdown. Scale bar, 10 μm. (**c**) FISH images of *Eleanor*-containing foci (green) obtained with the *ESR1*-BAC probe in LTED cells. The reduction of *Eleanor* foci by resveratrol was dependent on ER. Scale bar, 10 μm. (**d**) Box plot showing quantification of the FISH signals in **c**. *n*>200 nuclei for each sample. *P*-values were calculated using Student's *t*-test (***P<0.001). (**e**) Immunofluorescence analysis of ER degradation in LTED cells treated with ICI 182,780 (100 nM) for 48 h. Scale bar, 10 μm. (**f**) ICI 182,780 treatment inhibited the reduction of *Eleanor* foci by resveratrol treatment. The results were similar to those obtained by ER knockdown in **c**. Scale bar, 10 μm. (**g**) qRT–PCR analyses of LTED cells showing repression of *ESR1* mRNA by resveratrol treatment (RES +). ICI 182,780 had no de-repressive effect (RES +, ICI +). Values are the means±s.d.; *n*=3. *P*-values were calculated using Student's *t*-test (*P<0.05, ***P<0.001).

As described above, addition of resveratrol to LTED cells dramatically co-suppressed the expression of both *Eleanors* and *ESR1* mRNA (Figs 1b,e and 4b, red bars). As a result, resveratrol inhibited the proliferative activity of LTED cells in a dose- and time-dependent manner (Fig. 5a). Because oestrogen-loaded ER negatively regulates the *ESR1* gene^42^, we expected that resveratrol would repress the *ESR1* locus through ER. To test this hypothesis, we depleted ER with specific siRNAs in LTED cells followed by resveratrol treatment (RES, Fig. 5b). FISH analyses using the *ESR1*-BAC probe showed that the *Eleanor*-containing foci in control cells (si*GL3*) became smaller after resveratrol treatment, but they remained large in ER-depleted cells (si*ESR1*, Fig. 5c,d). These results indicated that suppression of *Eleanors* by resveratrol is dependent on ER. To confirm this result, we used a specific ER antagonist, ICI 182,780, which induces degradation of ER through the ubiquitin-mediated pathway^43^. After ICI 182,780 treatment of LTED cells for 48 h, ER was absent (Fig. 5e). Under this condition, the enlarged *Eleanor* FISH signals became insensitive to the suppressive action of resveratrol (Fig. 5f). Moreover, we found that ER degradation by ICI 182,780 in LTED-RES cells resulted in de-repression of *u-Eleanor* transcripts (Fig. 4b, blue bars), indicating that resveratrol inhibits *u-Eleanor* expression via oestrogenic effects on ER. This result may be explained by the presence of multiple oestrogen-response elements in the *u-Eleanor* region (Supplementary Fig. 5, marked with yellow). Because *u-Eleanor* is required for enhanced expression of intragenic *Eleanor* in LTED cells (Fig. 4f), it is possible that resveratrol represses *u-Eleanor* through ER, leading to a subsequent reduction of intragenic *Eleanor*. In addition, repression of *u-Eleanor* and *Eleanors* by resveratrol was abrogated by ICI 182,780, whereas *ESR1* mRNA remained repressed under the same condition (Fig. 5g). Thus, *Eleanors* were expressed even under repression of the *ESR1* gene, indicating that *Eleanors* do not simply represent nascent transcripts or by-products of *ESR1* mRNA.

Resveratrol has been reported to activate SIRT1, a member of the sirtuin family of NAD^+^-dependent deacetylases. Therefore, we examined whether SIRT1 is involved in the repressive effect of resveratrol on *Eleanors*. We depleted SIRT1 by siRNAs in LTED cells (Supplementary Fig. 6a,b) and then visualized *Eleanor*-containing foci by FISH. Similar to the control (si*GL3*), *Eleanor*-containing signals became smaller after resveratrol treatment under SIRT1 knockdown, suggesting that resveratrol represses *Eleanors* in the absence of SIRT1 (Supplementary Fig. 6c). In addition, overexpression of SIRT1 in LTED cells did not change the FISH signals (Supplementary Fig. 6d). Thus, the effect of resveratrol on the *ESR1* locus is unlikely to depend on SIRT1.

### A large chromatin domain is defined by a cluster of ncRNAs

**Figure 6:**
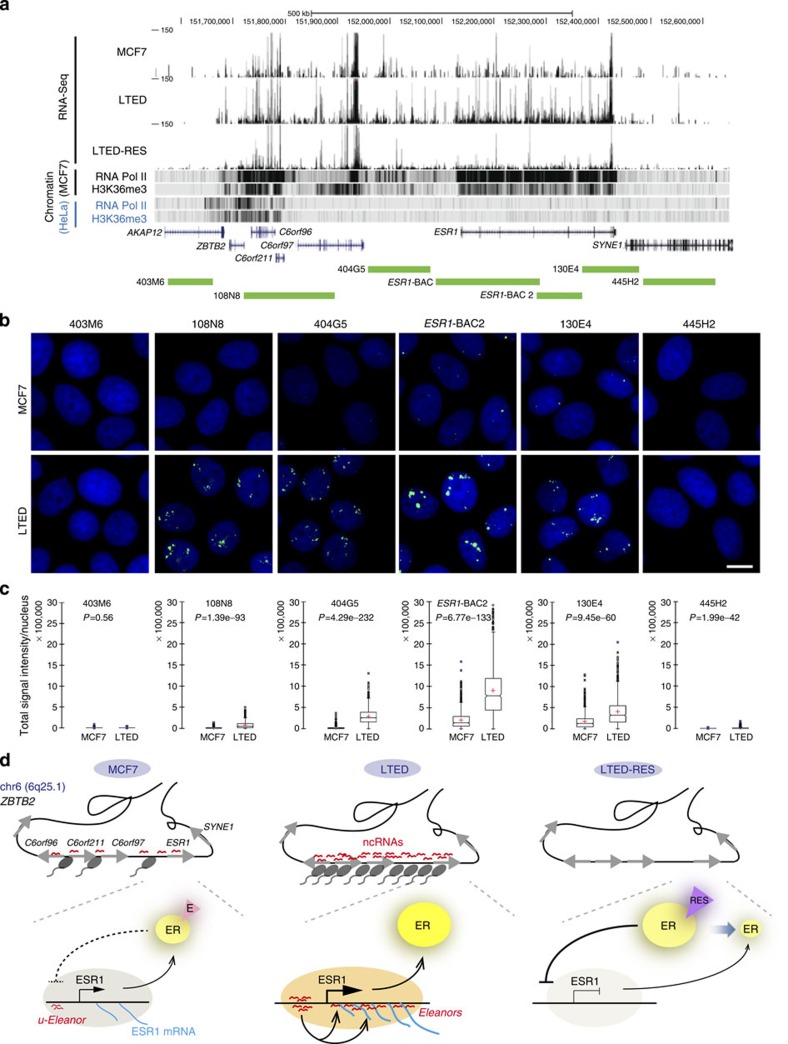
Large chromatin domain is defined by production of ncRNAs in LTED cells. (**a**) Overview of the ∼1 Mb region surrounding the *ESR1* locus. The RNA-Seq tracks were aligned with the ChIP-Seq data available in the UCSC genome browser^39^ (Supplementary Table 1). In LTED cells, RNAs were abundantly expressed from a large region including *C6orf96*, *C6orf211*, *C6orf97* and *ESR1* genes (6q25.1; 151,720,000–152,424,447). Green bars indicate the BAC clones used for FISH experiments. (**b**) RNA-FISH scanning analyses along the region of 6q25.1. Scale bar, 10 μm. In LTED cells, nuclear RNA foci were detected with BAC clones 108N8, 404G5, ESR-BAC2 and 130E4. (**c**) Box plot showing quantitative analysis of RNA-FISH in **b**. Total signal intensities per nucleus in MCF7 and LTED cells (*n*=700∼900 nuclei per sample). (**d**) Schematic model for ER-positive breast cancer adaptation. Overviews of the chromatin domain including the *ESR1* gene are at the top. RNA Pol II (a black oval with a tail) is bound to this region, and ncRNAs are transcribed at the basal level in MCF7 cells. In LTED cells, the ncRNAs are transcribed actively throughout the chromatin domain and stably associate with the site of transcription in LTED cells, leading to the formation of an RNA cloud in the nucleus. Resveratrol treatment suppresses the ncRNA production. Enlarged views of the *ESR1* locus are shown at the bottom.

An overview of the RNA-Seq data around the *ESR1* gene revealed that the region associated with ncRNAs spanned ∼700 kb (6q25.1; 151,720,000–152,424,447; Fig. 6a). In addition to *ESR1*, this region includes three previously annotated genes, *C6orf96*, *C6orf211* and *C6orf97*, all of which are co-regulated in breast cancer cells^44^. Alignment with the published ChIP-Seq data revealed that the region was heavily bound by RNA Pol II and enriched with an active histone mark, trimethyl-H3K36, in breast cancer cell lines, but not other cell types such as HeLa (Fig. 6a). The length of the region was well correlated with the recently proposed size for a single unit of a chromatin domain^19^,^20^,^21^.

To visualize characteristic RNAs originating from this large region, we performed RNA-FISH scanning using a series of BAC probes for subregions along the chromatin domain (Fig. 6a,b). We detected large RNA foci with probes 108N8, 404G5 and 130E4, which were similar to those detected with *ESR1*-BAC and *ESR1*-BAC2 probes (Figs 6b,c and 2b). It should be noted that 404G5, which corresponded to a region completely devoid of any protein-coding sequences, was able to detect the large RNA foci. In contrast, RNAs were not produced from outside of the domain (403M6 and 445H2). Using the combination of RNA-Seq and RNA-FISH, we found a novel chromatin domain including four co-regulated genes that were defined by induction of a ncRNA cluster during hormone deprivation (see Fig. 6d).

### A set of long genes exhibit co-regulation of ncRNA and mRNA

**Figure 7:**
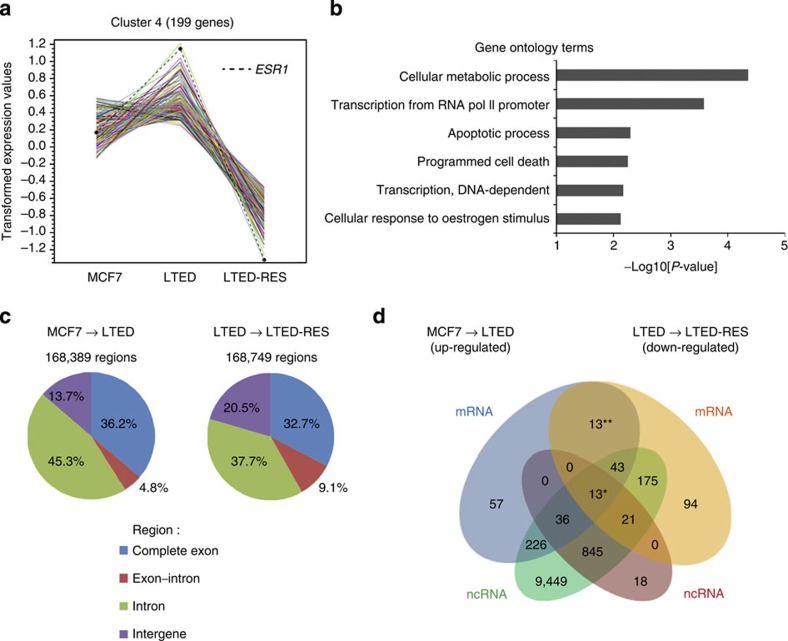
Genome-wide transcriptome analyses of LTED adaptation and resveratrol treatment. (**a**) Expression patterns of mRNAs from 199 genes in cluster 4. *ESR1* was the most dynamically up-regulated in LTED cells and down-regulated in LTED-RES cells. (**b**) Gene ontology terms significantly enriched in cluster 4. (**c**) Summary of RNA-Seq data in LTED adaptation (MCF7 to LTED) and resveratrol treatment (LTED to LTED-RES). Differentially expressed RNAs (log2 fold change <−1.0 or >1.0, *P*<0.01) were classified into four groups: complete exon, exon–intron, intron and intergene, as described in the Methods. *P*-value was calculated with edgeR^68^. The percentages of ncRNAs, excluding complete exons, were 63.8% (left) and 67.3% (right). (**d**) Genes with coordinate regulation of ncRNAs and mRNAs. Comparison of mRNA-Seq and ncRNA-Seq data resulted in the extraction of 13 gene regions (single asterisk) that showed simultaneous up-regulation of mRNAs and ncRNAs under MCF7 to LTED conditions and subsequent down-regulation under LTED to LTED-RES conditions (log2 fold change <−1.0 or >1.0, *P*<0.01). *P*-value was calculated with DESeq and edgeR^67^,^68^. The other 13 genes (double asterisks) also showed up- or down-regulation of mRNAs in LTED and LTED-RES cells, respectively, while ncRNAs showed no co-regulation. The genes in these groups and their gene lengths are listed in Table 1.

Our genome-wide transcriptome analyses revealed changes in the mRNA expression of 2,918 genes under MCF7, LTED and LTED-RES conditions (interquartile range >10). They were classified into 14 distinct clusters on the basis of expression patterns (Supplementary Fig. 7a). Among them, genes in cluster 4 (199 genes including *ESR1*) showed induction in LTED cells and repression in LTED-RES cells (Fig. 7a). Gene ontology analysis of cluster 4 showed enrichment of genes for the cellular metabolic process, transcriptional regulation, apoptosis and cell death, and cellular response to oestrogen stimulus (Fig. 7b).

We further characterized ncRNAs that were co-regulated with their neighbouring protein-coding genes. RNA-Seq analyses showed that ∼168,000 gene regions were significantly up- or down-regulated during LTED adaptation (MCF7 to LTED) and resveratrol treatment (LTED to LTED-RES, Fig. 7c). Interestingly, over 60% of these gene regions corresponded to potential ncRNAs, including exon–intron fusion, intron and intergenic sequences. We then extracted gene regions in which both mRNAs and ncRNAs were up-regulated during LTED adaptation and down-regulated by resveratrol treatment (Fig. 7d). Thirteen gene regions (indicated with a single asterisk in Fig. 7d and Table 1), including the *ESR1* locus, exhibited such coordinate expression in LTED and LTED-RES cells (Fig. 7d and Supplementary Fig. 7b). We found that the genes in this group were generally long. Their average length was close to 300 kb (280,142 bp), while the average for genes in the overall genome is ∼60 kb (ref. 45). In the other 13 gene regions (indicated with double asterisks in Fig. 7d and Table 1), similar to the *ESR1* locus, mRNAs were up- and down-regulated in LTED and LTED-RES cells, respectively. However, they lacked coordinated regulation with neighbouring ncRNAs (Fig. 7d and Supplementary Fig. 7c). Their average length was ∼17 kb (16,839 bp).

Thus, our comprehensive transcriptome analyses revealed that coordinated regulation of ncRNA and mRNA, as exemplified by *Eleanors* and *ESR1* mRNA, is common in a set of long genes. On the basis of recent studies^45^,^46^,^47^, ncRNA production from the entire gene locus may overcome physical transcription complications and maintain open chromatin.

## Discussion

In this study, we found a novel type of ncRNA-mediated gene locus control in breast cancer adaptation. While cells undergo hormone-independent proliferation, the *ESR1* gene is up-regulated and ncRNAs are produced from a broad chromatin domain of ∼700 kb including *ESR1* and other co-regulated genes. *Eleanors* originate from and around the *ESR1* gene, and maintain the transcriptionally active locus. Up-regulation of *ESR1* is important for the hormone-independent cell growth, which is suppressed by inhibition of *Eleanors* with either *u-Eleanor* knockdown or resveratrol. *Eleanors* are overexpressed in ER-positive breast cancers, suggesting that resveratrol and an inhibitor of *u-Eleanor* may be potential therapeutic agents for endocrine therapy resistance.

On the basis of our data, we propose a mechanistic model as illustrated in Fig. 6d. *ESR1* mRNA is expressed at the basal level in MCF7 cells as they are ER positive (Fig. 6d, MCF7). During LTED adaptation, because of the loss of negative control by oestrogen-bound ER, there is significant induction of *u-Eleanor* expression, leading to coordinate up-regulation of intragenic *Eleanor* and *ESR1* mRNA (Fig. 6d, LTED). Characteristic *Eleanor*-containing RNA foci are formed in the nuclei of LTED cells and subsequent resveratrol treatment represses both *u-Eleanor* and intragenic *Eleanor* by the oestrogenic actions on ER (Fig. 6d, LTED-RES).

The mechanism for induction of *u-Eleanor* in LTED cells is intriguing, and one possible mechanism is de-repression. In MCF7 cells, oestrogen-bound ER mildly inhibits the *ESR1* locus by negative feedback^48^. On removal of oestrogen, ER becomes unliganded and fails to repress the *ESR1* locus. By the addition of resveratrol to LTED cells, resveratrol-bound ER strongly represses the *ESR1* locus, possibly because of its high concentration or structural properties.

Cancer cells can survive during various environmental changes by adjusting their global gene expression to acquire suitable phenotypes. Indeed, our transcriptome analyses of MCF7 and LTED cells showed that 2918 mRNAs were significantly up- or down-regulated under oestrogen deprivation (Supplementary Fig. 7a). These transcriptional changes were not limited to protein-coding regions (Fig. 7c, complete exon) and rather prevalent (∼2-fold more frequent) in noncoding regions (Fig. 7c, exon–intron, intron and intergene). These results are in good agreement with the fact that <2% of the mammalian genome encodes proteins, whereas 75% of the genome is transcribed to produce ncRNAs that may modulate chromatin structure and gene expression^26^,^49^.

For the following reasons, *Eleanors* identified in this study are unique. First, transcription of *Eleanors* is inducible. They accumulate during oestrogen deprivation, before *ESR1* mRNA up-regulation, and are abruptly suppressed by resveratrol. Furthermore, genome-wide analyses revealed that the *ESR1* locus is one of the 13 genetic loci where ncRNAs and mRNAs are co-regulated during the hormonal changes.

Second, intragenic *Eleanors* are transcribed from a broad region covering the entire *ESR1* gene body, which spans up to 300 kb. In addition, *u-Eleanor* is derived from an enhancer-like element at ∼40 kb upstream of the *ESR1* gene. *u-Eleanor* is responsible for up-regulation of downstream transcripts including *ESR1* mRNA and intragenic *Eleanors* (Fig. 4d,f). To date, there are few reports of simultaneous transcription of ncRNAs from upstream and the gene body. One related but distinct example is the β-globin locus where ncRNAs are produced from LCR, a central upstream regulatory element, and downstream intergenic regions in the locus^50^.

Third, *Eleanors* cover their own transcription sites to regulate gene expression and form a so-called ‘RNA cloud' in the nucleus (Fig. 2c). This property may be shared with other chromosomal RNAs including *XACT*, *Air*, C_0_T-1 repeat RNAs and snoRNAs^51^,^52^,^53^,^54^. *XIST* RNA also forms a large nuclear domain by coating the entire inactive X chromosome and plays a role in X chromosome inactivation^55^. *Eleanors* may be functional introns that are stably maintained in the nucleus^56^,^57^,^58^ or represent pervasive transcription of the genome^59^, which are enhanced under oestrogen deprivation. *Eleanors* were unusually stable because of resistance to the denaturation procedure that is normally performed only in DNA FISH and degrades most RNAs. Because the *Eleanor* FISH signals were clearer with denaturation (Fig. 2b, top panels), *Eleanors* may be tethered to the sites of transcription by RNA-DNA hybrid formations.

We found that coordinate expression of ncRNA and mRNA is conserved in a set of long genes (Fig. 7, Table 1). Accumulation of ncRNA at the site of transcription may counteract length-dependent impairment of gene transcription that was reported recently^45^,^47^. We and others have previously reported that a population of ER-positive breast cancer cells has abnormally large *ESR1* FISH signals^60^,^61^,^62^. Considering that *Eleanors* are extremely stable, it is possible that some of the previously detected large FISH signals in breast cancer patients include *Eleanors* as shown in Fig. 3. In addition, several molecular events have been reported during LTED adaptation, including up-regulation of ER, ERBB2, c-Myb, c-Myc and MAP kinases, and activation of the PI-3 kinase pathway, NOTCH pathway, growth factor pathways related to mTOR and EGFR/ERBB/AKT, as well as changes in the phosphorylation pattern of ER^7^,^8^,^10^,^31^,^63^. It would be interesting to determine how *Eleanors* are integrated in these events in LTED cells and ER-positive breast cancer cells.

In summary, *Eleanors*, a novel type of ncRNA, are actively involved in the epigenetic adaptation of ER-positive breast cancer cells by activating transcription of the *ESR1* gene locus. These findings highlight ncRNA-mediated mechanisms in cancer cell adaptation, which may be diagnostic and therapeutic targets for endocrine therapy-resistant breast cancer.

## Methods

### Cell culture

MCF7 cells (ATCC) were cultured in RPMI 1640 (Sigma) supplemented with 10% fetal bovine serum (FBS). For LTED, MCF7 cells were grown in phenol red-free RPMI 1640 (Wako) containing 4% dextran-coated charcoal-stripped FBS for 2–4 months. For LTED-RES, LTED cells were treated with 50 or 100 μM resveratrol (Sigma Aldrich, R5010) for 24 h. For ICI 782,780 treatment, cells were cultured with 100 nM ICI 782,780 (Tocris, 1047) for 48 h. Human mammary epithelial cells^64^ (Lonza) were cultured in mammary epithelial growth media (Lonza) according to manufacturer's protocol at 37 °C with 5% CO_2_. Primary invasive breast carcinoma specimens were obtained by surgical excision from patients at the Department of Breast and Endocrine Surgery, Kumamoto University Hospital (Kumamoto, Japan).Informed written consent was obtained from all the patients before surgery. The study protocol was approved by the Ethics Committee of Kumamoto University Graduate School of Medicine (Kumamoto, Japan).

### Antibodies

The following primary antibodies were used: rabbit polyclonal anti-human ERα (Santa Cruz Biotechnology, sc-543; dilution used in IB: 1:1,000, IF: 1:300), rabbit monoclonal anti-human ERα clone SP1 (Ventana, 790–4325; used in IHC without dilution), mouse monoclonal anti-human β-tubulin (Sigma Aldrich, T4026; dilution used in IB: 1:1,000), anti-RNA polymerase II (phosphor-S5) (Abcam, ab5131; dilution used in ChIP: 1:200), histone H3K4me1 (Abcam, ab8895; dilution used in ChIP: 1:200), histone H3K4me3 (Millipore, 07–473; dilution used in ChIP: 1:200), rabbit IgG (Santa Cruz Biotechnology, sc-2027; dilution used in ChIP: 1:100), rabbit polyclonal anti-SIRT1 (Millipore, 07–131; dilution used in IB: 1:1,000, IF: 1: 200), and mouse monoclonal anti-myc (Roche, clone 9E10 dilution used in IB: 1:1,000, IF: 1:100). The secondary antibodies used were Alexa Fluor 488-conjugated donkey anti-rabbit IgG (Molecular Probes; dilution used in IF: 1:300) and Cy3-conjugated donkey anti-mouse IgG (Jackson ImmunoResearch; dilution used in IF: 1:1,000). FITC-anti-digoxigenin (Roche; dilution used in FISH: 1:250) or Cy3-streptavidin (Jackson ImmunoResearch; dilution used in FISH: 1:1,000).

### BAC clones

For FISH analysis, we used BAC probes covering the *ESR1* locus and flanking regions (RP11-403M6, RP11-108N8, RP3-404G5, RP11-450E24 (for *ESR1*-BAC), RP1-63I5 (for *ESR1*-BAC2), RP1-130E4 and RP3-445H2) and a probe containing the *ERBB2* locus (RP11-94L15). To verify primers used in the 3C assay, we used BAC clones spanning the *ESR1* locus (RP11-450E24 and RP3-404G5).

### Preparation of mRNA-Seq libraries

Total RNA was extracted from cultured cells with an RNeasy Mini Kit (Qiagen). mRNA-Seq libraries were generated using an mRNA-Seq Sample Preparation Kit (Illumina) according to manufacturer's protocol with minor modifications. Poly(A)^+^ RNA was enriched from 1 μg total RNA by two successive rounds of oligo(dT) selection, fragmented and then used for first-strand cDNA synthesis by random hexamer priming. After second-strand cDNA synthesis, double-stranded DNA was repaired using T4 DNA polymerase, Klenow enzyme and T4 polynucleotide kinase (New England Biolabs), and then treated with Klenow exo- to add an adenine to the 3′ end. After ligation of the Index PE Adaptor oligo mix (Illumina) using Takara ligation mix (Takara), the adaptor-ligated DNA was amplified using primers, InPE2.0/1.0 and index (Illumina), by 18 cycles of PCR. The amplified libraries were isolated from an agarose gel. The DNA samples were purified using a QIAquick MinElute Kit (Qiagen) at each preparation step.

### Preparation of RNA-Seq libraries

Total RNA was extracted from cultured cells with an RNeasy Mini Kit and then ribosomal RNA was removed by a Ribo-Zero Gold Kit (Illumina). RNA-Seq libraries were generated using a ScriptSeq v2 RNA-Seq Library Preparation Kit (Illumina), according to manufacturer's instructions.

### mRNA-Seq and RNA-Seq analyses

An Illumina Genome Analyzer IIx was used to generate 41-base single-end reads for mRNA-Seq and an Illumina HiSeq 1000 was used to generate 51 base (stranded) single-end reads for RNA-Seq. The sequence reads were aligned with the human reference genome (UCSC hg19, http://genome.ucsc.edu/) using Tophat (v1.4.1, ref. 65), and RNA transcripts were reconstructed with Cufflinks (v1.3.0, ref. 66) with all parameters set to default values. The numbers of total and mapped reads are summarized in Supplementary Table 2.

### Clustering of sequence reads and gene ontology

Data for mRNA-Seq experiments were analysed using CLC Genomics Workbench ver 5.51 (CLC Bio). Results obtained from mRNA-Seq experiments were selected on the basis of the following parameters: degree of variance in gene expression (inter quantile range >10) in two samples among MCF7, LTED and LTED-RES cells, and sufficient abundance of mRNA (FPKM >5). A total of 2,918 genes were chosen and used for downstream analyses including k-means clustering (Mev (http://www/tm4.org/men/)). In addition, cluster 4 (199 genes) obtained by k-means clustering was used for gene ontology analysis that was performed using the Genomatix Pathway System in the Genomatix Genome Analyzer. The distribution of reads in the RNA-Seq data set was determined by counting the number of reads in a 100-bp sliding window by the NGS Analyzer of the Genomatix Genome Analyzer and selected on the basis of the following parameters: differentially expressed transcripts in two different cell states (MCF7 and LTED, or LTED and LTED-RES) by fold-change ranking (log2 fold-change <−1.0, down-regulation; >1.0, up-regulation) together with *P*-values (*P*<0.01) computing by method described in chapter ‘Testing for differential expression' in DEseq algorithm version 1.0.6 (ref. 67) in the NGS Analyzer of the Genomatix Genome Analyzer. This analysis identified 168,389 and 168,749 genetic regions that were differentially expressed in LTED cells compared with that in MCF7 cells, and LTED-RES cells compared with that in LTED cells, respectively. Furthermore, each region was subdivided by ElDorado database genome annotation version 08–2011.

### Combined expression analysis of mRNA and ncRNA

Genetic regions corresponding to mapped read clusters in exon–intron junctions, introns and intergenes were detected using the NGS Analyzer and defined as ncRNA candidates (Fig. 7c). Moreover, read clusters with different levels between LTED and MCF7 cells, and LTED-RES and LTED cells were extracted using edgeR1.6.15 in the Genomatix Genome Analyzer (Fig. 7d). Genetic regions that were up-regulated in LTED cells compared with that in MCF7 cells were selected on the basis of fold-change ranking (log2 fold-change <−1.0) together with *P*-values (*P*<0.01), which identified 388 mRNAs and 10,808 ncRNAs (Genomatix Software GmbH). *P*-values were calculated by edgeR algorithm version 1.6.15 (ref. 68). Similar selections were performed for down-regulated mRNAs and ncRNAs in LTED-RES cells compared with that in LTED cells, resulted in 359 mRNAs and 933 ncRNAs. Overlapping among the four groups was analysed and summarized to show the number of shared genes in a four-way Venn diagram (Genomatix Software GmbH).

Nonspecific enrichments detected in each sample were subtracted from the local enrichments (clusters) representing genomic regions bound in comparative samples.

### ChIP-Seq data used in this study

ChIP-Seq data of MCF7 cells were obtained from ENCODE Consortium through UCSC genome browser (University of California, Santa Cruz), and compared with our mRNA-Seq and RNA-Seq data (Figs 4a and 6a and Supplementary Figs 3a and 4a). The files used in the analyses are listed in Supplementary Table 1.

### Fluorescence *in situ* hybridization

Cells grown on coverslips were fixed with 4% formaldehyde and 0.5% Triton X-100 in phosphate-buffered saline (PBS) for 15 min, and then permeabilized with 0.5% saponin and 0.5% Triton X-100 in PBS for 20 min. Samples were immersed in 20% glycerol in PBS for 30 min, and then subjected to four cycles of freeze–thawing by freezing the cells in liquid nitrogen for 30 s each time. The cells were then treated with 0.1 N HCl for 15 min. For denaturation and hybridization, the cells were incubated in hybridization mixtures (2 × SSC, 50% formamide, 10% dextran sulfate, 1 mg ml^−1^ tRNA and 5–10 μg ml^−1^ probe DNA) at 75 °C for 4–10 min and then 37 °C for 48–72 h. BAC and plasmid probes were labelled with biotin or digoxigenin in a nick translation mixture (Roche) according to the manufacturer's protocol. After hybridization, the cells were washed with 2 × SSC and 50% formamide at 37 °C for 5 min, followed by 2 × SSC at 37 °C for 5 min. FISH signals were detected with FITC-anti-digoxigenin (Roche; dilution 1:250) or Cy3-streptavidin (Jackson ImmunoResearch; dilution 1:1,000). For dual-colour FISH, a SPEC *ESR1/CEN6* Dual Color Probe kit (ZytoVision) was used. For nuclease treatment, cells were pre-treated with 0.5 U μl^−1^ DNase I (Promega) or 1 μg ml^−1^ RNase A (Roche) for 30 min before hybridizations.

For FISH with tissue microarray (BioChain, Z7020005 and Biomax, BR1504), sections were processed using ZytoLight SPEC ESR1/CEN 6 Dual Color Probe Kit (Zytovision, Bremerhaven, Z-2070-20) according to manufacturer's protocol. Briefly, slides were heated for 10 min at 70 °C, treated with xylene twice for 10 min at room temperature, rehydrated in a series of graded ethanol solutions, washed with dH_2_O, incubated in Pretreatment Solution Citric at 98 °C for 15 min and then washed again with dH_2_O. The slides were then treated with pepsin solution for 10 min at 37 °C, and washed with 2 × SSC solution for 5 min and with dH2O for 1 min at room temperature. The slides were dehydrated, air-dried and incubated with a probe at 76 °C for 10 min for denaturation, and at 37 °C overnight for hybridization. DNA was counterstained with 5,6-diamidino-2-phenylindole (DAPI).

### Immunofluorescence and immunohistochemistry

For immunofluorescence, cells were fixed with 4% paraformaldehyde in PBS for 15 min at room temperature. The cells were washed three times with PBS for 5 min, then permeabilized with PBS containing 0.2% Triton X-100 and 0.5% normal goat serum (GIBCO) for 5 min on ice. The cells were incubated at room temperature with PBS with 0.5% normal goat serum (GIBCO) three times for 5 min, then with the primary antibodies for 60 min, followed by the Cy3- or Alexa488-conjugated secondary antibodies for 60 min. The cells were washed with PBS three times for 10 min each. DNA was counterstained with 1 μg ml^−1^ DAPI.

For immunohistochemical staining, sections in a tissue microarray (Biomax, BR1504) were processed with automated IHC staining system, BenchMark XT (Ventana). The sections were de-paraffinized in EZ prep (Ventana) at 72 °C for 4 min and then incubated in Immunoblock (DS Pharma Biomedicals) at 37 °C for 12 min to block endogenous peroxidase. Antigens were retrieved by incubating 95 °C for 8 min, then treated with rabbit monoclonal anti-human ERα clone SP1 (Ventana, 790–4325, used without dilution) at 37 °C for 36 min. The antigens were visualized by avidin–biotin-peroxidase complex method using iVIEW DAB detection kit (Ventana). The slides were then counterstained with haematoxylin at 37 °C for 8 min.

### Microscopy and image analysis

Images were obtained with an IX-71 microscope (Olympus) equipped with a × 60 NA1.0 Plan Apo objective lens, a cooled CCD camera (Hamamatsu) and image acquisition software (Lumina Vision Version 2.4; Mitani Corporation). For imaging cytometry analyses of FISH, image stacks of three-dimensional data sets were collected at 0.5–1.0 μm intervals through the *z* axis, subjected to projections and used for automatic detection and counting of FISH signals with CELAVIEW RS100 software (Olympus) and Cellomics CellInsight (Thermo Fisher Scientific). Images in Figs 1c and 2c and Supplementary Fig. 1c were obtained with a confocal laser-scanning microscope (LSM 780, Carl Zeiss) equipped with × 63/1.4 Plan-Apochrome objective lens and a cooled CCD camera (Carl Zeiss). Image acquisition was done using LSM software (Carl Zeiss).

### Immunoblotting

To prepare total cell lysate, cells were dissolved in SDS sample buffer containing benzonase (Sigma). Proteins were separated by SDS–polyacrylamide gel electrophoresis and then transferred to a nitrocellulose membrane, Amersham Hybond ECL (GE Healthcare). The membrane was blocked for 1 h with PBS containing 10% nonfat dry milk and then incubated with primary antibodies in PBS containing 0.03% Tween 20 for 1 h. The membrane was washed with PBS containing 0.3% Tween 20 three times for 10 min each, and incubated with horseradish peroxidase-conjugated secondary antibodies for 40 min. After the membrane was washed with PBS containing 0.3% Tween 20 three times for 10 min each, signals were visualized with Western Lightning Plus-ECL (PerkinElmer). Uncropped immunoblot images for Figs 4e and 5b, and Supplementary Fig. 6a are provided in Supplementary Figs 8 and 9, respectively.

### PCR with reverse transcription

Total RNA was isolated from cultured cells with TRIzol (Invitrogen) and then treated with DNase I (Roche) before cDNA synthesis. Reverse transcription was carried out with a High Capacity cDNA reverse transcription kit (Applied Biosystems). qRT–PCR was performed with SYBR green fluorescence on an ABI Prism 7300 system (Applied Biosystems). Values were normalized to β-actin or glyceraldehyde-3-phosphate dehydrogenase (*GAPDH*) gene expression before calculating relative fold changes. The amplification efficiency for each primer set was measured using genomic DNA and used for normalization in Figs 2a and 4b. To indicate absolute expression levels of each RNA in Figs 2a,e and 4b, ΔC_t_ values are summarized in Supplementary Table 6. Primer sequences are listed in Supplementary Table 3. For RT–PCR, amplified products were separated by electrophoresis on 2% agarose gels and stained with ethidium bromide. All PCR amplifications were performed within a quantitative range by adjusting the cycle numbers. For RT–PCR in Supplementary Fig. 3b, PCR-amplified products were run on agarose gel and stained with ethidium bromide. Corresponding uncropped gel images are provided in Supplementary Fig. 9.

### Chromatin immunoprecipitation-qPCR

MCF7 and LTED cells were crosslinked with 1% formaldehyde at 37 °C for 10 min. Crude cell lysates were sonicated to generate DNA fragments of 200–500 bp using a Bioruptor USD-250 (Cosmo-Bio; 15 sonications of 30 s with 30 s intervals at 250 W). Chromatin was precipitated with antibodies at 4 °C overnight, washed and de-crosslinked for 5 h. DNA enrichment in ChIP samples was determined by qPCR with SYBR green fluorescence on an ABI Prism 7300 system. The threshold was set to cross a point where PCR amplification was linear, and the cycle number required to reach the threshold was recorded and analysed using Microsoft Excel. PCR was performed using precipitated DNA and the input DNA. Primers used in ChIP-qPCR are listed in Supplementary Table 3.

### RNA interference

Cells were transfected with specific siRNAs (Nippon EGT) using RNAiMAX (Invitrogen). Target sequences for each siRNA are listed in Supplementary Table 3. The cells were analysed at 48 and 72 h after transfection.

### Cell counting

The number of proliferating cells was counted using an automatic cell imaging counter (CYTORECON; GE Healthcare) or image cytometer (Cellomics CellInsight; Thermo Fisher Scientific).

### 3C assay

Formaldehyde-crosslinked chromatin from MCF7 and LTED cells was digested with *Bgl*II restriction enzyme overnight, followed by ligation with T4 DNA ligase at 16 °C for 4 h. To prepare control templates for standard curves, a BAC clone covering the *ESR1* locus (RP11-450E24 and RP3-404G5) was digested with *Bgl*II, followed by random religation. After reversing the cross-links, genomic DNA was purified by phenol extraction and ethanol precipitation. The ligated products were assessed by qPCR with the ABI Prism 7300 and Thunderbird SYBR qPCR Mix (Toyobo). The efficiency of *Bgl*II digestion was evaluated by qPCR using primers that only amplified undigested DNA fragments containing the *Bgl*II site. More than 80% of the individual restriction sites were digested under the experimental condition. 3C-qPCR data were normalized to a loading control that was obtained with primers that amplified a genomic fragment in the *ESR1* locus (intron 3), which represented the amount of template DNA. Primers used in the 3C assay are listed in Supplementary Table 3.

### Overexpression of SIRT1 in LTED cells

Full-length cDNA for human SIRT1 was amplified by PCR using primers described in Supplementary Table 3. The amplified fragments were digested with *Eco*RV and *Xba*I, and then cloned into pcDNA3-*myc* (pcDNA3-*myc*-SIRT1). LTED cells (1 × 10^5^ cells) were transfected with the plasmid (2 μg) using 5 μl FuGene HD (Roche Applied Science) in a six-well plate for 48 h, and then subjected to immunofluorescence or immuno-FISH.

### Statistical analysis

Comparisons between groups were analysed using the two-tailed Student's *t*-test. A value of *P*<0.05 was considered statistically significant. For RNA- and mRNA-Seq experiments, *P*-values were calculated with DESeq and edgeR^67^,^68^.

## Author contributions

S.T., M.O.A.A., N.S. and M.N. designed and conducted the experiments, and prepared the manuscripts, together with support from S.F., H.M. and H.I. K.M. and Y.O. conducted and analysed high-throughput sequencing experiments.

## Additional information

**Accession codes:** mRNA-Seq and RNA-Seq data sets have been deposited in the DNA Data Bank of Japan (DDBJ) Sequence Read Archive (accession number: DRA001006).

**How to cite this article:** Tomita, S. *et al.* A cluster of noncoding RNAs activates the *ESR1* locus during breast cancer adaptation. *Nat. Commun.* 6:6966 doi: 10.1038/ncomms7966 (2015).

## Supplementary Material

Supplementary Information Supplementary Figures 1-9 and Supplementary Tables 1-6

## Figures and Tables

**Table 1:** Gene sets with or without coordinate regulation of ncRNAs and mRNAs.

Genes with coordinated transcription*		**Genes without coordinated transcription**†	
**Gene symbol**	**Gene length (bp)**	**Gene symbol**	**Gene length (bp)**
*SDK1*	967,552	*ERBB2*	40,523
*LRBA*	750,839	*BRD8*	38,900
*RERE*	465,236	*SLC12A5*	38,461
*ESR1*	412,778	*CLN3*	25,430
*USP34*	283,260	*SIDT2*	18,223
*SYNPO2*	210,560	*DBNDD1*	14,659
*KDMA2A*	138,811	*CASP14*	8,813
*KYNU*	111,912	*ESRP2*	7,687
*AVL9*	88,604	*TUBG2*	7,759
*CCDC50*	68,586	*CYP1A1*	5,995
*SYTL2*	63,780	*VPS28*	4,963
*AHNAK*	41,104	*APH1A*	3,811
*ATIC*	37,818	*MFSD10*	3,677
Average length	280,142	Average length	16,839

^*^Genes that showed simultaneous up-regulation of mRNAs and ncRNAs under MCF7 to LTED conditions and subsequent down-regulation under LTED to LTED-RES conditions (denoted with single asterisk in Fig. 7d).

^†^Genes that showed up- and down-regulation of mRNAs in LTED and LTED-RES cells, respectively, while ncRNAs showed no co-regulation (denoted with double asterisks in Fig. 7d).
