# Supplementary material for: A cluster of noncoding RNAs activates the ESR1 locus during breast cancer adaptation
Source: Nat Commun. 2015 Apr 29;6:6966. doi: 10.1038/ncomms7966 (PMC4421845; doi:10.1038/ncomms7966)
Supplement: Supplementary Information — Supplementary Figures 1-9 and Supplementary Tables 1-6 [file ncomms7966-s1.pdf]

Supplementary Information

A cluster of non-coding RNAs activates the *ESR1* locus during breast cancer adaptation

Saori Tomita, Mohamed Osama Ali Abdalla, Saori Fujiwara, Haruka Matsumori, Kazumitsu Maehara, Yasuyuki Ohkawa, Hirotaka Iwase, Noriko Saitoh, and Mitsuyoshi Nakao

Supplementary Information contains:

Supplementary Figures 1-9

Supplementary Tables 1-6

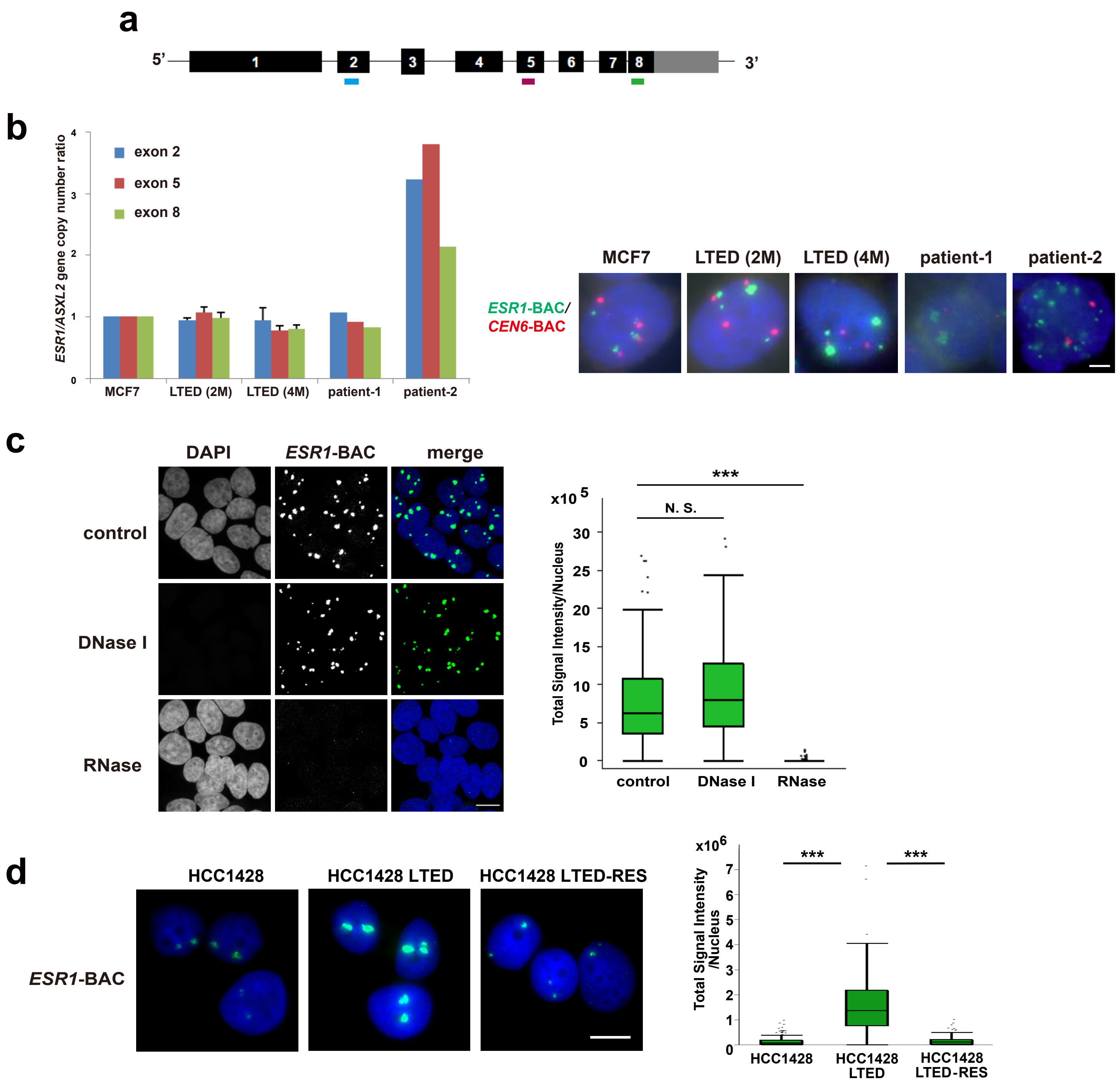

Supplementary Figure 1. Enlarged FISH signals are mainly composed of RNAs

(a) Schematic diagram of the *ESR1* gene. Black boxes with numbers represent exons. Positions for primers used in (b) are denoted with colored bars at the bottom. **(b)** Copy number variation analysis. Relative copy numbers of the *ESR1* gene were measured by genomic qPCR (left). Values for MCF7 cells were set to 1. Each value was normalized to the *ASXL2* gene. Corresponding FISH images (right). Relative copy numbers were constant in MCF7 and LTED cells, and patient-1 samples, suggesting that *ESR1* gene amplifications did not occur in LTED cells. In contrast, patient-2 samples showed significant *ESR1* amplification (>2.0) that corresponded to numerous scattered FISH signals. Bar, 10 μm. **(c)** FISH signals were enriched with RNAs. The enlarged FISH signals were detected in LTED cells using the *ESR1*-BAC probe (green, control). The signals were not affected by DNase treatment (DNase I), but showed a marked decrease in size by RNase treatment (RNase A). DNA was stained with DAPI. Genomic DNA was completely digested by the DNase. The residual FISH signals after RNase treatment represented *ESR1* locus DNA. Thus, the FISH signals were mainly composed of RNAs transcribed from the *ESR1* locus. Right panel shows quantification of the FISH signals. **(d)** RNA-FISH of breast adenocarcinoma cell line HCC1428, probed with *ESR1*-BAC. *Eleanor* FISH signals were enlarged with estrogen deprivation for 1 month (HCC1428 LTED), and suppressed with 100 μM resveratrol for 24 h (HCC1428 LTED-RES). Right panel shows quantification of the FISH signals. In (c) and (d) : bar 10 μm, n > 150 nuclei/sample, P-values were calculated using Student' s *t*-test (***p<0.0001, N. S., not significant).

a

Gene	Expression value in MCF7 mRNA-Seq (RPKM)	Expression value in LTED mRNA-Seq (RPKM)	Fold Change (LTED / MCF7)	Chromosome position
ESR1	52.21811	102.8718	1.97004	6
ERBB2	18.60553	37.56174	2.018848	17
APP	71.97262	114.2807	1.587836	21
ERGIC1	60.11627	119.1596	1.982153	5

b

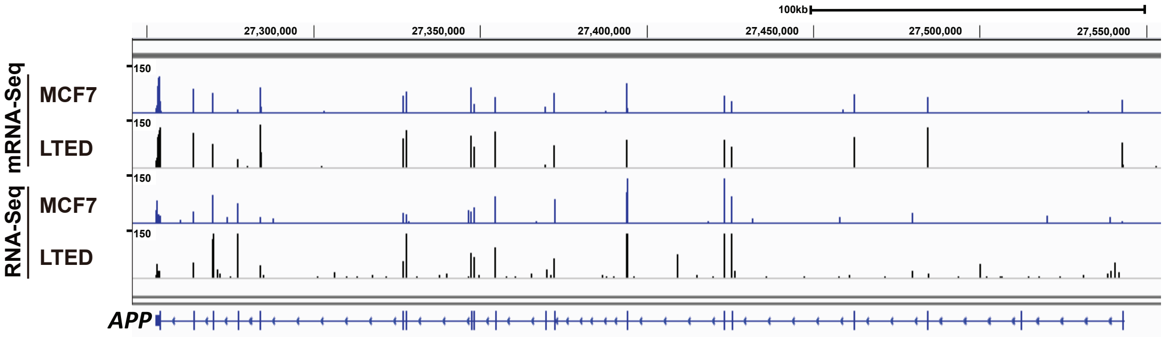

c

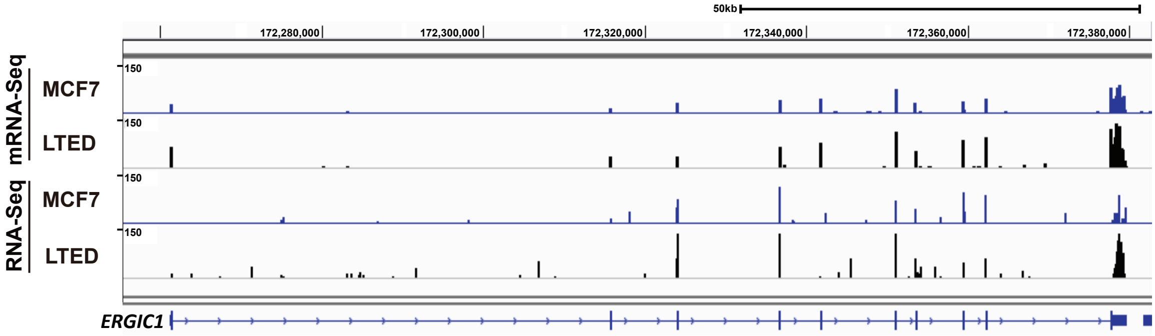

d

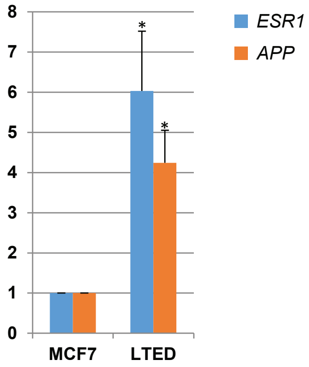

Supplementary Figure 2. Genes that are up-regulated without ncRNA transcription in LTED cells
(**a**) Genes that were up-regulated in LTED; *APP* (*amyloid beta (A4) precursor protein*), *ERBB2*, *ERGIC1* (*endoplasmic reticulum-golgi intermediate compartment*) and *ESR1*. Each expression value derived from the mRNA-Seq is shown. All of the four genes showed the similar level of fold change (approximately 2.0) in LTED relative to MCF7 cells. (**b** and **c**) Gene tracks representing mRNA-Seq and RNA-Seq of the *APP* (b) and *ERGIC1* (c) loci. Unlike the *ESR1* locus shown in Fig. 1e, there was no induction of ncRNAs in these loci in LTED cells. (**d**) qRT-PCR experiment shows comparable induction levels of *ESR1* and *APP* mRNAs in LTED cells. Values in MCF7 were set to 1. Values are the means ± SD; n = 3. P-values were calculated using Student's *t*-test (*$p<0.05$).

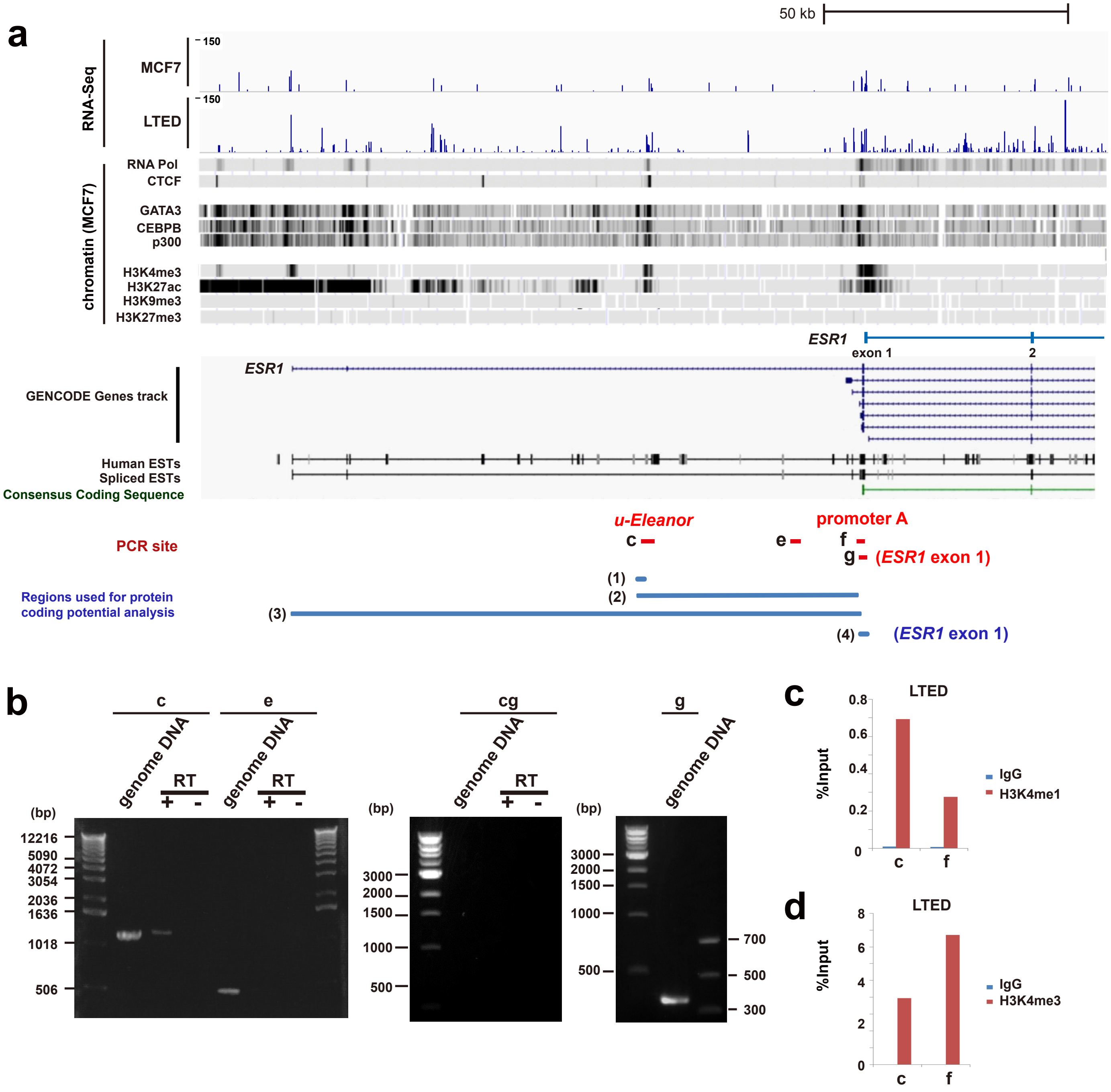

Supplementary Figure 3. *u-Eleanor* is transcribed from a region upstream of the *ESR1* gene
(**a**) Overview of a region upstream of the *ESR1* locus. The RNA-Seq tracks were aligned with the ChIP-Seq data available in the UCSC genome browser (**Supplementary Table 1**), gene annotation from GENCODE version 19, human expressed sequence tags (ESTs) localization in GenBank, gene annotation from the Consensus Coding Sequence project. Sites for PCR experiments in **Supplementary Fig. 3b**, **c** and **d** (c, e, f and g), and regions used for prediction of protein coding potential by CPAT program (http://lilab.research.bcm.edu/cpat/) in **Supplementary Table 5** ((1) ~ (4)) are shown. Regions g and (4) correspond to *ESR1* exon 1. (**b**) Expression of *u-Eleanor* from site c in LTED cells. In agreement with the results in **Fig. 4b**, RT-PCR experiments detected transcription at site c, but not site e. A transcript of ~1200 nucleotides corresponded to a part of *u-Eleanor*. A transcript that links *u-Eleanor* and promoter A (cg) was not detected, excluding the possibility that *u-Eleanor* is an alternative promoter. Genomic DNA was successfully amplified with the same primers in site g (g), confirming the feasibility of the primers. Total RNA was treated with DNase prior to reverse transcription (+RT). (**c** and **d**) ChIP-qPCR analyses of H3K4me1 (c) and H3K4me3 (d). The levels of modified histone H3 were measured in the *u-Eleanor* gene region (site c) and *ESR1* promoter A (site f). The *u-Eleanor* region was marked with H3K4me1 rather than H3K4me3.

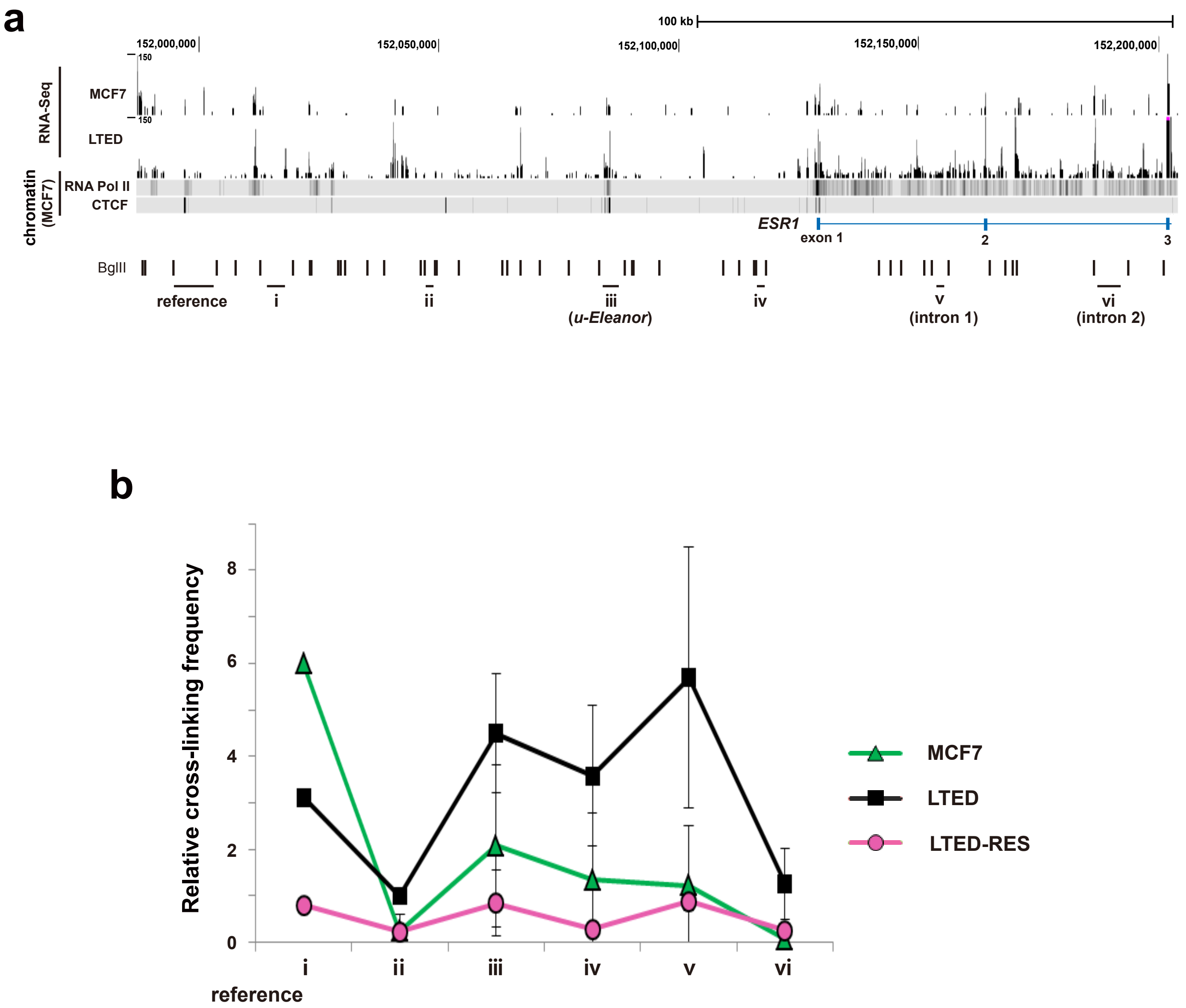

Supplementary Figure 4. Chromatin conformation changes around the *u-Eleanor* gene region during LTED adaptation

(a) Overview of a region upstream of the *ESR1* locus. The RNA-Seq tracks were aligned with the ChIP-Seq data available in the UCSC genome browser (**Supplementary Table 1**). Positions of BglII sites and the analyzed fragments (reference and i–vi) are shown at the bottom. **(b)** Relative chromosomal interactions between the reference and distal sites (i–vi) were measured by chromosome conformation capture (3C)-qPCR. The interaction frequency between the reference and site ii in LTED cells was set to 1. Each value was normalized to qPCR values obtained with BAC templates. To compare MCF7, LTED and LTED-RES cells, the values obtained with the internal control primers (*ESR1* intron 3) were used as a DNA loading control. Values are the means ± SD; n = 3.

(-45218)

GTCACCTTTAAGGGGCCTCTTTAAATCCCAGCTCTTTCATTTACAAATTCTATGACCTTGGATGAGTTTTTCACTTTT

CTGTGCCACAAGTTCTCAGTAAAATGGAGATAATTATAGAACTTACTTCATAGAGCTTTGAGAATCATACAGGTAAAG

TTATTTTACGTAATTAGCACTCAAAAGTGACCATTAATAATCTAACGTTTTAAAATATATTAATAGGCCAGATGCAGT

GGCTCACACCTGTAATTTCAGCACTTTGGGAGGCTGAAGTGGGTGGATCACTTGAGGTCAGGGGTTCAAAGCCAGCCT

GGCCAACATGGCGAAACTCCGTCCCTACTAAAACTGTAAAAATTAGCTGGGCATAGTGGCGTGTGTCTGTAGTCCCAG

CTATTCAGGAGGCTGAGGCAGGAAAATCGCAGTGAGCCGAGATCACGCCACTGCACTCTAGCCTGGGTGACAGAGTGG

GACTCCATCTCAAAAACAAACAAACAAACAAAAATTAATAGTTTAAATATTTTTAATATATTTCAATATATAAAGATG

TAATTTTTATTTTAAATATATTAAAATAGATAAGTTTAAAATACATAAAGTAAATATATAAAATTAATATGTTAAAAT

GTTTACTGAAAGTTTACATTTTTCTCTGTATTGCTAAGAGCAATTTATTGCCTTATATGTTGTGTAATATCTAGAAAA

CACACCTATGTAATTTCCAAATTATAACCACCTTGGCTCCTGGACAAGTAGGACTGTGTCTGATTTTTTTTTTTGCAT

CCTTGTCACTACCTGCCACTCTGAGATCACTTGTTGTCTTTCCATATATAATCACCTCTAGGTACACTTCTTTCATTA

TTTCTACGCATCCTCTCCACTTTGCTGCATTCTTGTGTGCATTCTTTTCTCCCACGTTGGAAAGAAATATGCATATTG

AGCGATTCCAACCCCCAAAGGCAAAAATTACTAGCCAGTGTTGAAAATGAAAGCAAGGAATTTTTTGGTTATTGTTGC

TGTAACTTTTTGATGTGCGTACATATGTGCGTCTTTTTTTTCCCCCTAGGGGTGGAATGGGGTGGTGTTAGTCAGGGA

TTACTGACAGGACGGTGTTGCCTACTGTGTTGGAGAGACTAAGACTGGGGAAAGATTCTCCAAGGCTGGGAAATGCAG

ACGTCCAGTCTTGGAAAACAGTTTGTCGTTGGTCGTTATGTCAGAAGAGGAGTGTATTCCTGGAAGAAGGCCCACAAA

ATATACCCAGAGCCCAGGTGCCTTTCTAAGACGAATCAACATTCACTCACAAGTTCAGCTAATATATGACAGAAATAC

AAGAATGAACAAGACTCCTGACTTAAGCTTACAGCTGATGGGAAACTTGGGAACTGGACAGAGAATTAAAATACAACG

TGGTTAGGTACAACTTACCTGCGCCAGAGGAGTGACGGAAGGCCTTGGACGGGGAGTGATGCCTGGAGGATGACAGGG

CGACAGGTAAGTTAGGGGGCTGGGAAGGGGAAGGGCGGCCCTGTGGGTAAAGGGAAGGAGGTTAAAGTCAGCAGGGAA

ACAGGCAGTTCCCTGTTGCTGGAGCAAAGCGCGGGGGTGTGGCTCTCCTGTCTCTGGCTTTGCTGTCAACAGGCGAGA

TTGGCAAGTAGGGCGACCAACCGGCCTGGTTTGCTCGGGACCGGGGTTTCCTGGAAGGTGGGACTTTCAATGCTAAAA

CTGGGACAGTCCTGGGCAAACAAATCAGTACGGTTGGCCACCCTATCTTTGTGTGGGCATCTGCTAACTGAGTCATGG

GCCTGTCTGGTTTATTTATTTATTTTTTTTCTGCCTGAGTAACAGAAGAATGTGTTAAACATATGGAGTATGGAAGAA

GAAGGAGAGGGGAGATAGGATTTTTATGTTATAGATTGGAAGAAGACATCCTTGATGAAGGAGATGTTTTTACTTTGG

AGAAGGACTGCCTCAGCCGCGAAAGACCAAGGGGTGGCACCACAGGGAGAAGTCTGCACTGCGCTTGTCAGGACGGCC

AGTGGGGGGCTGCTGAGCGCAGGGAGTCAGTCCTCGCAATGAAGATATAGTCTCGGGGAGATAATGCTGGAAATCGAT

GGAAGAAGTGGAACAGGACACAAATTTAGTGGAATTTCTTTACAATGTGTGCTTCTTGTCACCAGTTCGTTAACCATG

ATAAAAACGGTTATGTTGTTACATTCATCAAGATCCAAATTTTCTACCATTTTAAGATGTCTATCAGTCACAATGATG

ATCACTGAAGACCGAACGAGATCATTTCCTCTAAAAATTATGTTACATTTATCCATAATGGCATCAGTGTCTGTTACA

CGTTTGCTTTAATTTTGAGGTCAAATACGACATTTGTAAATCTAGCACTTCATGTGATTCTGTAAGCACTAAACAGCT

AAACATATTTACTTCTTTTTTTTGAATCAATTAGAACAGTGCTGTACAATAGAAAAATAGAACAGTGCTGTACAATGG

AAACAGTGCTGTACAACAGAAATCTGTCAGCCACACGTGTCGTTTAAACTTTTCTAGTAATCACATTAAAATTGTAAA

AAGAAGCCAATAAAAGTAATTTTAA (-42619)

Supplementary Figure 5. Putative estrogen-response elements are present in the *u-Eleanor* gene region
DNA sequence around the *u-Eleanor* region. Sites c#1 (red) and c#2 (green) in the *u-Eleanor* region were analyzed in Figure 4. Putative ER binding sites (yellow) were predicted by Weblogo (http://weblogo.berkeley.edu/). Nucleotide numbers indicate positions from the *ESR1* transcription start site.

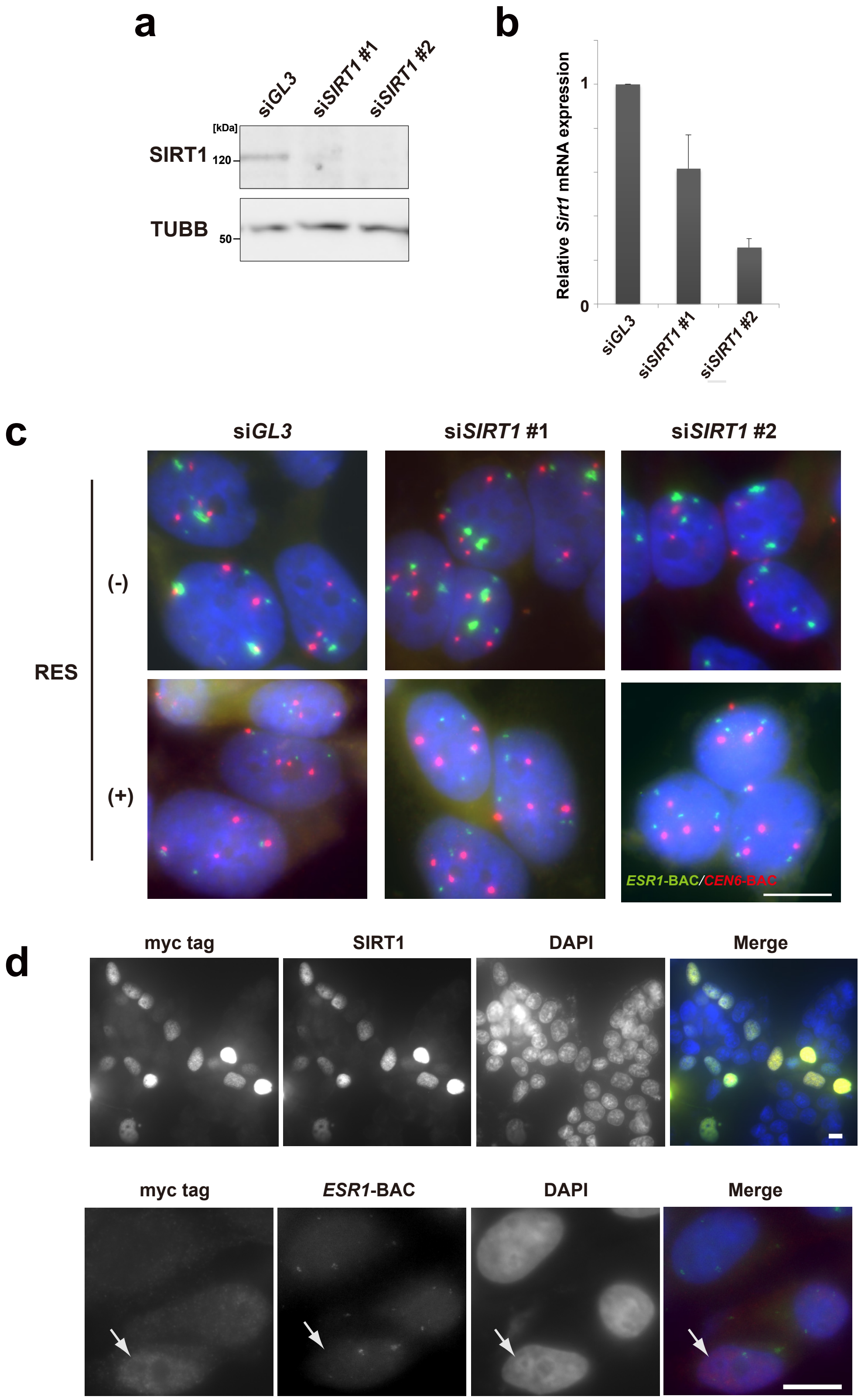

Supplementary Figure 6. **Repressive effects of resveratrol on the *ESR1* locus are independent of SIRT1**
(a and b) siRNA-mediated knockdown of SIRT1. Immunoblotting (a) and qRT-PCR (b) were carried out with LTED cells. Values are the means ± SD; n = 3. **(c)** SIRT1 knockdown did not affect the suppressive effect of resveratrol on FISH signals. FISH analyses of heat-denatured samples showed *Eleanor*-containing foci (green, *ESR1*-BAC probe) and centromeric regions (red, *CEN6*-BAC probe) in LTED cells. Bar, 10 µm. **(d)** No effect of SIRT1 overexpression on FISH signals. In the upper panels, immunofluorescence analyses of LTED cells expressing myc-tagged SIRT1 were performed using antibodies against myc-tag (red) and SIRT1 (green), together with DAPI staining (blue). In the lower panels, immuno-FISH analyses showed that SIRT1-overexpressing LTED cells (red, arrows) maintained the enlarged *Eleanor*-containing foci (green, *ESR1*-BAC probe) compared with that in adjacent control cells. Bar, 10 µm.

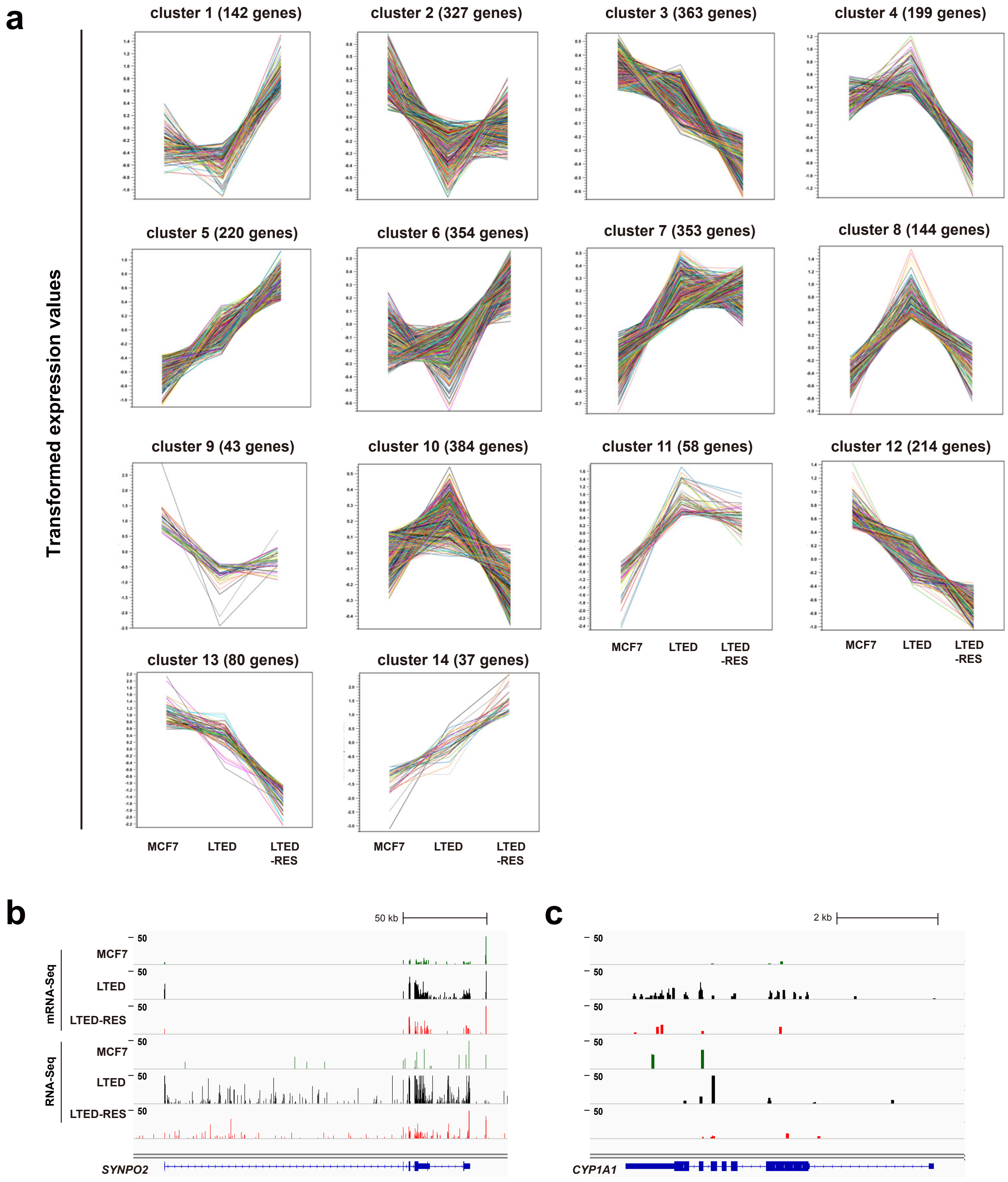

Supplementary Figure 7. Expression patterns of genes that are differentially regulated in MCF7, LTED, and LTED-RES cells

(a) The 2,918 genes that underwent dynamic changes in mRNA expression were classified into 14 clusters using a k-means clustering algorithm. **(b** and **c)** Representative gene loci that showed up-regulation of mRNA in LTED cells and down-regulation in LTED-RES cells. The *SYNPO2* locus showed coordinate expression of ncRNA and mRNA in LTED and LTED-RES cells (b), while the *CYP1A1* locus did not show such expression (c).

Figure 4e

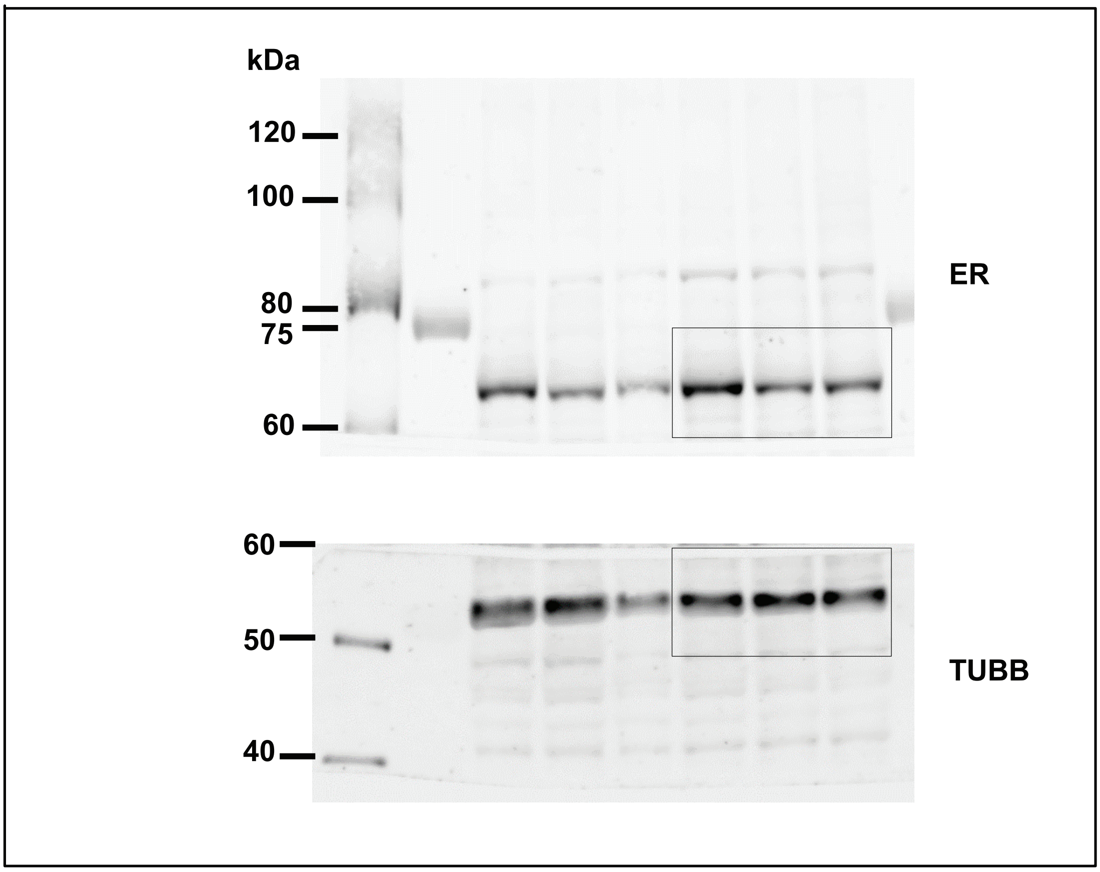

Figure 5b

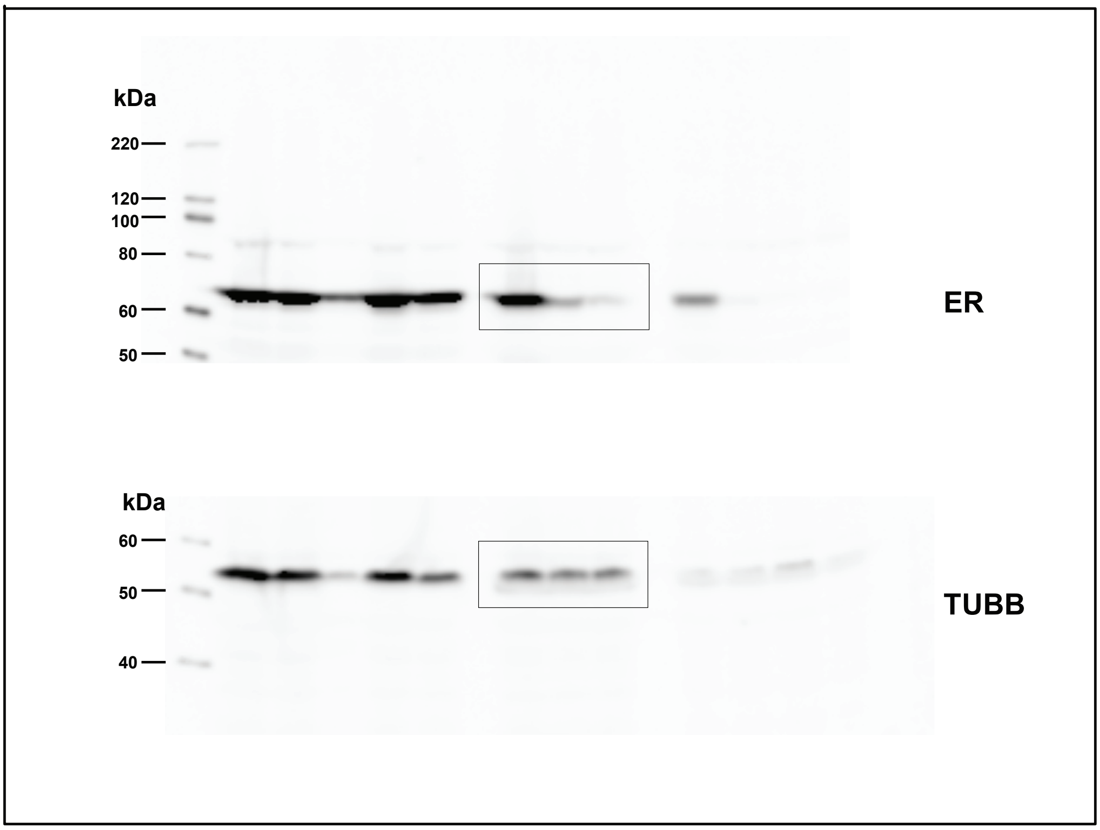

Supplementary Figure 8. Uncropped immunoblots for Figs 4e and 5b

Supplementary Figure 3b

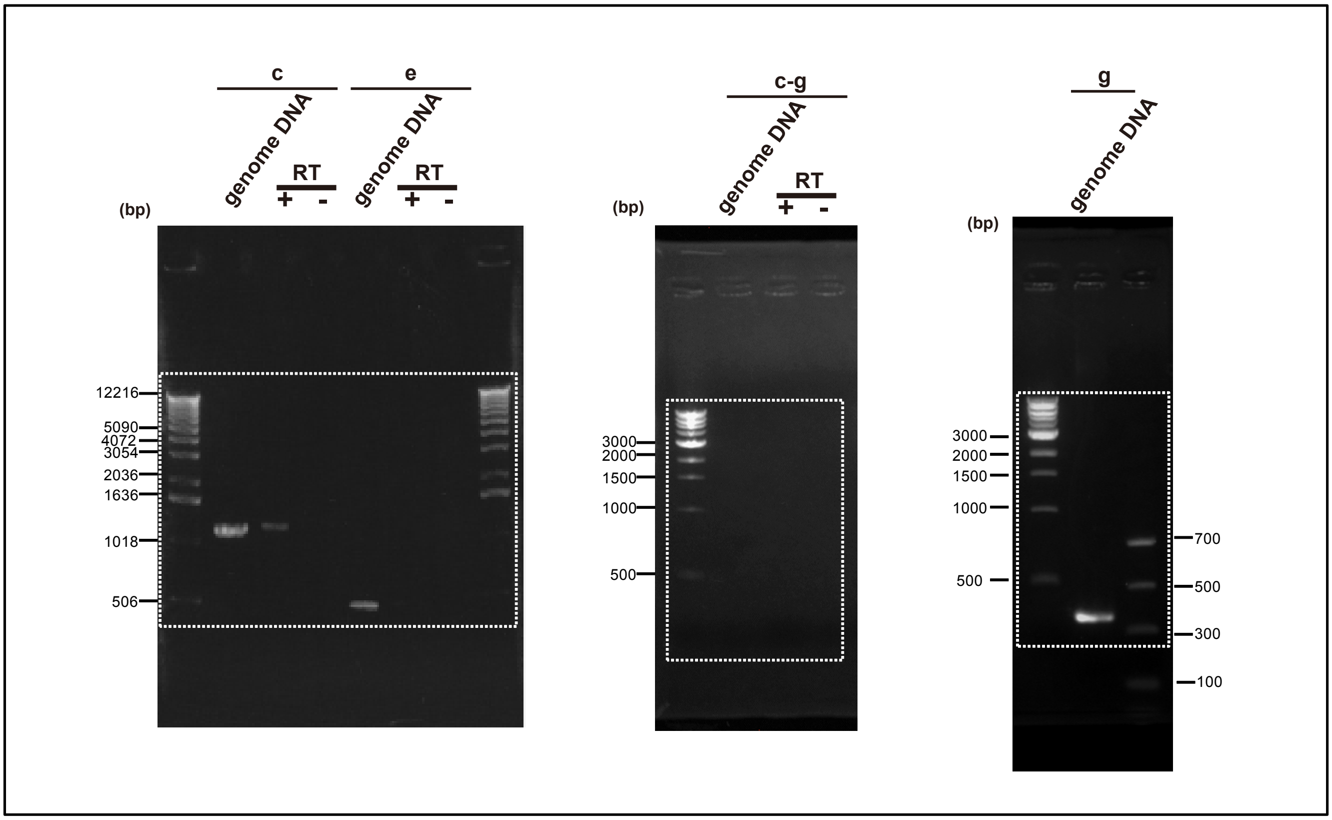

Supplementary Figure 6a

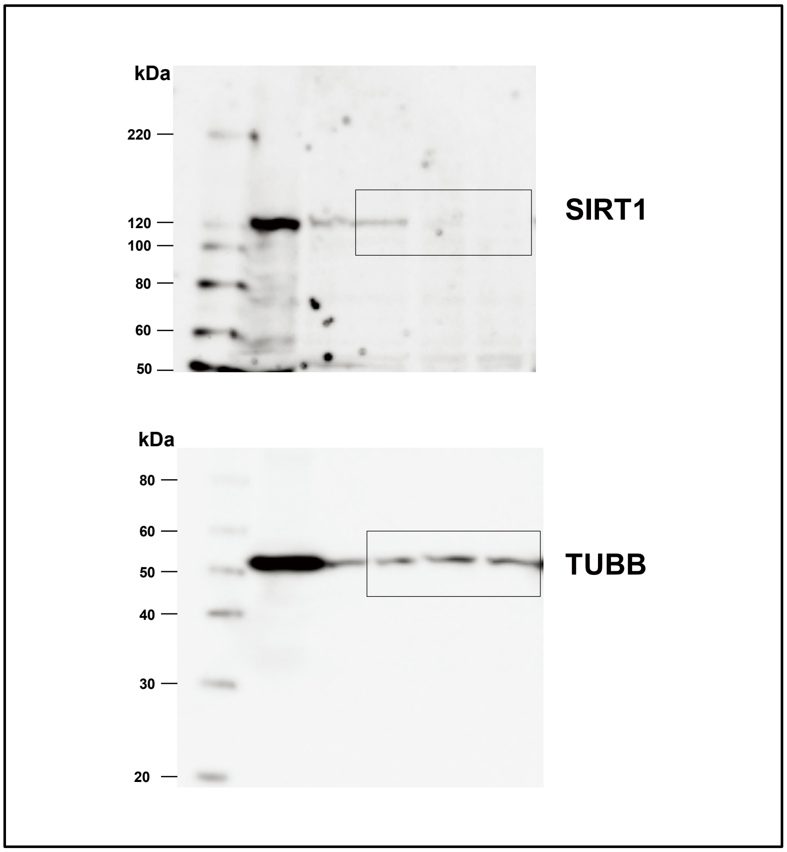

Supplementary Figure 9. Uncropped agarose gel and immunoblots for Supplementary Figs 3b and 6a

Supplementary Table 1. ChIP-Seq data used in this study

Data	File Name
RNA Pol II<br>(Crawford, Iyer-UT Austin)	wgEncodeOpenChromChipMcf7Pol2Sig.bigWig<br>wgEncodeOpenChromChipHelas3Pol2Sig.bigWig
CTCF<br>(Crawford, Iyer-UT Austin)	wgEncodeOpenChromChipMcf7CtcfSig.bigWig
GATA3<br>(Myers, Hudson Alpha)	wgEncodeHaibTfbsMcf7Gata3V0422111RawRep1.bigWig
CEBPB<br>(Myers, Hudson Alpha)	wgEncodeHaibTfbsMcf7Cebpbsc150V0422111RawRep1.bigWig
p300<br>(Myers, Hudson Alpha)	wgEncodeHaibTfbsMcf7P300V0422111RawRep1.bigWig
H3K4me3<br>(Stamatoyannopoulous, UW)	wgEncodeUwHistoneMcf7H3k4me3StdRawRep1.bigWig
H3K27ac<br>(Snyder, Farnham-USC)	wgEncodeSydhHistoneMcf7H3k27acUcdSig.bigWig
H3K9me3<br>(Snyder, Farnham-USC)	wgEncodeSydhHistoneMcf7H3k09me3UcdSig.bigWig
H3K27me3<br>(Snyder, Farnham-USC)	wgEncodeSydhHistoneMcf7H3k27me3bUcdSig.bigWig
H3K36me3-MCF7<br>(Snyder, Farnham-USC)	wgEncodeSydhHistoneMcf7H3k36me3bUcdSig.bigWig
H3K36me3-HeLa<br>(Bernstein, Bernstein - Broad Institute)	wgEncodeBroadHistoneHelas3H3k36me3StdSig.bigWig

Supplementary Table 2. Mapped reads in mRNA/RNA Seq

	sample	total	unmapped	mapped	mapped(%)
mRNA Seq	MCF7	36,067,192	7,158,285	28,908,907	80.15%
	LTED	31,122,231	8,857,592	22,264,639	71.54%
	LTED-RES	25,651,739	5,234,428	20,417,311	79.59%
RNA Seq	MCF7 (stranded)	24,740,764	13,047,062	11,693,702	47.27%
	LTED (stranded)	34,817,960	21,456,745	13,361,215	38.37%
	LTED-RES (stranded)	41,471,290	20,511,699	20,959,591	50.54%

Supplementary Table 3. Primers and siRNAs

qPCR primer	Sequence
ESR1 No.1-mRNA-S	5'-AGAACGAGCCCAGCGGCTAC-3'
ESR1 No.1-mRNA-AS	5'-CCTTGCAGCCCTCACAGGAC-3'
ESR1 No.2-mRNA-S	5'-TTACTGACCAACCTGGCAGA-3'
ESR1 No.2-mRNA-AS	5'-ATCATGGAGGGTCAAATCCA-3'
ESR1 exon 1-S	5'-ATGACCCTCCACACCAAAGCAT-3'
ESR1 exon 1-AS	5'-ATCTTGAGCTGCGGACGGTT-3'
ESR1 intron 2 site-a-S	5'-TTCAGAAGGTTGTGTGGCGTAGA-3'
ESR1 intron 2 site-a-AS	5'-CAGCTTCTGTTGGCACCTTA-3'
ESR1 intron 2 site-b-S	5'-CCATTGCTGAGTTAGCTGC-3'
ESR1 intron 2 site-b-AS	5'-AAACGGCTTAGATGAAGGAG-3'
ESR1 intron 2 site-c-S	5'-GTTTCACTGTGTTGGGCACACTA-3'
ESR1 intron 2 site-c-AS	5'-ATTTTAAAACGAGCCAGGCA-3'
ESR1 intron 2 site-d-S	5'-TAGTAGAGGCTGTTCCCTTC-3'
ESR1 intron 2 site-d-AS	5'-CTGTAACCTTACCCATAGCC-3'
ESR1 intron 2 site-e-S	5'-TCATGAGCAATGCGTGGCTT-3'
ESR1 intron 2 site-e-AS	5'-CAAGGCTTGGTTCTAAGCAG-3'
ERBB2 intron 11-S	5'-CATATGGGGAGCACTGTCTG-3'
ERBB2 intron 11-AS	5'-TTACTGCCACCTGCTCCACA-3'
ESR1 5'UTR-a-S	5'-AAGAATGTCTGATGGTGCCCTG-3'
ESR1 5'UTR-a-AS	5'-CAAGTAGAACTCCAGAGTGAG-3'
ESR1 5'UTR-b-S	5'-GAAGTCTCTTCTCGAATGCT-3'
ESR1 5'UTR-b-AS	5'-TCTCAAGTCCCGTATCTTCA-3'
ESR1 5'UTR-c#1-S	5'-GAATGAACAAGACTCCTGAC-3'
ESR1 5'UTR-c#1-AS	5'-TCTGGCGCAGGTAAGTTGTA-3'
ESR1 5'UTR-c#2-S	5'-CTAGCACTTCATGTGATTCT-3'
ESR1 5'UTR-c#2-AS	5'-CCATTGTACAGCACTGTTCT-3'
ESR1 5'UTR-d-S	5'-TTTGAAGCACCCCTCTCTTCCT-3'
ESR1 5'UTR-d-AS	5'-GTCCTTAGTACCTCGTTCCT-3'
ESR1 5'UTR-e#1-S	5'-GCTCCCCAGAGAGAAATTAA-3'
ESR1 5'UTR-e#1-AS	5'-GCCTATCACTACAGAAGACA-3'

ERBB2-mRNA-S	5'-CCTCACAGAGATCTTGAAAG-3'
ERBB2-mRNA-AS	5'-AGAGCGGTTGGTGTCTATCA-3'
ESR1 exon 2-S	5'-TCTGCCAAGGAGACTCGCTACT-3'
ESR1 exon 2-AS	5'-CACAGGACCAGACTCCATAATGG-3'
ESR1 exon 5-S	5'-TTGACCCTCCATGATCAGGTC-3'
ESR1 exon 5-AS	5'-ACGAGACCAATCATCAGGA-3'
ESR1 exon 8-S	5'-CAACATCAGCAGTAAAGTCC-3'
ESR1 exon 8-AS	5'-TACGGCAAGCTAGGCAATGG-3'
ASXL2-S	5'-CCTGAGCCTTGAAGATTCT-3'
ASXL2-AS	5'-ACTGATAAGAGAGGCCTCTGA-3'
Sirt1-mRNA-S	5'-AGCTCTAGTGACTGGACTC-3'
Sirt1-mRNA-AS	5'-ATGATTGGCACAGATCCTCG-3'
GAPDH-mRNA-S	5'-ACACCCACTCCTCCACCTTT-3'
GAPDH-mRNA-AS	5'-TAGCCAAATTCGTTGTCATACC-3'
**ChIP-qPCR primer**	**Sequence**
ESR1 5'UTR-c#3-S	5'-ACTTTGGAGAAGGACTGCCT-3'
ESR1 5'UTR-c#3-AS	5'-TTCATTGCGAGGACTGACTC-3'
ESR1 5'UTR-f-S	5'-TTCGTCCTGGGACTGCACTTG-3'
ESR1 5'UTR-f-AS	5'-AAAAGAGCACAGCCCGAGGTTA-3'
**PCR primer**	**Sequence**
u-Eleanor-c#4-S	5'-GACATCCTTGATGAAGGAGA-3'
u-Eleanor-c#4-AS	5'-GCATGGCTAACGAGAGTAAC-3'
ESR1 5'UTR-e#2-S	5'-AACCCTTGCCCAGCAAATCA-3'
ESR1 5'UTR-e#2-AS	5'-AGATCCACCACACAGCTGAT-3'
ESR1-exon1-g-S	5'-ATCAGATCCAAGGGAACGAG-3'
ESR1-exon1-g-AS	5'-TCCAGGTAGTAGGGCACCTG -3'
APP-S	5'-GCCAAGAAGTCTACCCTGAA-3'
APP-AS	5'-AGTTCTGGATGGTCACTGGTT-3'
**3C primer**	**Sequence**
reference	5'-GTCATCATGTAGTCCTGGGTCT-3'
i	5'-CGTTCTCCAAACTGATGACCA-3'
ii	5'-TTGGCTCAAGCCAAGTAGATG-3'

iii	5'-GCATACCTAATCAGCTAATTCTG-3'
iv	5'-ATGAGTCCTAGCCTATGCTC-3'
v	5'-GGAAACAAAGTCATTACCTC-3'
vi	5'-GTGCCAGGATATTTTGAAAA-3'
loading control-S	5'-TAACCTGCTCTGACTCGCCT-3'
loading control-AS	5'-CAGTCTTTCTGGCACTGTATTC-3'
**siRNA**	Target sequence
u-Eleanor #1	5'-GGGAAGGAGGUUAAAGUCA-3'
u-Eleanor #2	5'-GCUUCUUGUCACCAGUUCG-3'
Sirt1 #1	5'-CUUGUACGACGAAGACGAC-3'
Sirt1 #2	5'-GCAACAGCAUCUUGCCUGA3'
GL3	5'-CUUACGCUGAGUACUUCGA-3'

Supplementary Table 4. *ESR1* FISH signals in breast cancer tissues

	ESR1 signal type				total sample number
	– [d]	–/+ [e]	+ [f]	++ [g]	
luminal type [a]	5 (9.3%)	5 (9.3%)	4 (7.4%)	40 (74%)	54
ERBB2 type [b]	48 (72.7%)	18 (27.3%)	0 (0%)	0 (0%)	66
triple negative type [c]	15 (75%)	4 (20%)	1 (5%)	0 (0%)	20
total	68	27	5	40	140

[a] Breast cancers containing HR (hormone receptor)+ ERBB2- type and HR+ ERBB2+ type, where HR+ indicates ER and/or PR (progesterone receptor) positive.

[b] Breast cancers of HR- ERBB2+

[c] Breast cancers of HR- ERBB2-

[d] no detectable signal

[e] faint signals

[f] median signals

[g] strong enlarged signals

Supplementary Table 5. Protein coding potentials predicted by CPAT program (http://lilab.research.bcm.edu/cpat/)

Region in Supplementay Fig.3a	Chromosomal position (RNA size)	Open reading frame size (bp)	Ficket Score	Hexamer Score	Coding Probability	Coding Label
(1)	chr6:152,084,924-152,086,091 (1,168)	261	0.7478	-0.2616481	0.00880203	no
(1)	chr6:152,083,595-152,086,194 (2,600)	261	0.7478	-0.2616481	0.00807495	no
(1)	chr6:152,084,000-152,086,000 (2,001)	261	0.7478	-0.2616481	0.00837155	no
(2)	chr6:152,084,000-152,129,000 (45,001)	441	0.7534	-0.0662936	0.0157989	no
(3)	chr6:152,011,000-152,129,000 (118,001)	477	0.6815	-0.2367289	7.29E-05	no
(4)	chr6:152,129,000-152,131,000 (2,001)	819	1.1702	0.3541562	0.99870944	yes

Supplementary Table 6. Mean ΔCT values of qRT-PCR experiments in Fig. 2a, 2e, and 4b

For Fig. 2a

exon1		a		b		c		d		e		ERBB2	
MCF7	3.49378	MCF7	4.8871	MCF7	4.71378	MCF7	4.43755	MCF7	4.36595	MCF7	4.4381	MCF7	9.34465
LTED	2.01553	LTED	3.0597	LTED	2.99485	LTED	2.56123	LTED	2.61155	LTED	2.65818	LTED	7.78683
RES	5.18195	RES	5.68048	RES	5.91428	RES	5.1647	RES	5.31305	RES	5.29723	RES	9.86785

For Fig. 2e

MCF7		LTED	
ESR1	5.59	ESR1	2.42
ERBB2	7.83	ERBB2	4.97

For Fig. 4b

a		b		c#1		c#2		d		e	
MCF7	15.0615	MCF7	12.7664	MCF7	11.6586	MCF7	11.7532	MCF7	13.8653	MCF7	17.6394
LTED	13.9258	LTED	9.61377	LTED	9.6708	LTED	9.87387	LTED	11.7876	LTED	15.0048
RES	14.4976	RES	12.1984	RES	12.2751	RES	11.453	RES	13.9229	RES	16.4617
ICI	12.8113	ICI	10.8151	ICI	9.98347	ICI	9.96257	ICI	12.5884	ICI	14.5272
